# Different Motile Behaviors of Human Hematopoietic Stem versus Progenitor Cells at the Osteoblastic Niche

**DOI:** 10.1016/j.stemcr.2015.09.003

**Published:** 2015-10-08

**Authors:** Katie Foster, François Lassailly, Fernando Anjos-Afonso, Erin Currie, Kevin Rouault-Pierre, Dominique Bonnet

**Affiliations:** 1 Haematopoietic Stem Cell Laboratory, The Francis Crick Institute, London WC2A 3LY, UK

## Abstract

Despite advances in our understanding of interactions between mouse hematopoietic stem cells (HSCs) and their niche, little is known about communication between human HSCs and the microenvironment. Using a xenotransplantation model and intravital imaging, we demonstrate that human HSCs display distinct motile behaviors to their hematopoietic progenitor cell (HPC) counterparts, and the same pattern can be found between mouse HSCs and HPCs. HSCs become significantly less motile after transplantation, while progenitor cells remain motile. We show that human HSCs take longer to find their niche than previously expected and suggest that the niche be defined as the position where HSCs stop moving. Intravital imaging is the only technique to determine where in the bone marrow stem cells stop moving, and future analyses should focus on the environment surrounding the HSC at this point.

## Highlights

• HSC niche should be defined by the position where HSCs stop moving
• HSC anchorage to the niche can be studies using time-lapse intravital imaging

## Teaser

In this article, Bonnet and colleagues dissect the early stages of human HSC anchorage to the niche using time-lapse imaging and demonstrate that HSCs show differential motile behaviors compared with their HPC counterparts when arriving in their niche.

## Introduction

Coordinating the balance between hematopoietic stem cell (HSC) quiescence and self-renewal is crucial for maintaining lifelong hematopoiesis and is controlled by a complex network of intrinsic and extrinsic signaling interactions with the microenvironment. While our understanding of the regulators controlling mouse hematopoietic stem/progenitor cells (HSPCs) has increased (reviewed in Morrison and Scadden, 2014), little is known about whether these factors and cellular micro-environmental component(s) that are important for mouse HSPCs could also be extrapolated to human HSPCs. The most widely used system that mimics the human niche in vivo is the xenotransplantation model. In this system, immunodeficient mouse bone marrow (BM) provides efficient support of human HSPCs allowing multilineage reconstitution. Once transplanted, HSPCs are home to the BM where they reside in specific niches that direct proliferation, quiescence, apoptosis, and mobilization into the periphery. Reconstitution can be followed by peripheral blood sampling or BM aspiration weeks after transplantation, but the first and most critical stages of lodgment (defined as their position at early time points post-transplant; Lapidot et al., 2005) are not well characterized. A recent study provided the first demonstration of the use of human-mouse xenografts as a surrogate model to study positioning of human HSPCs in human bone biopsy specimens, indicating that similar micro-environmental niches could be defined in the xenotransplant model (Guezguez et al., 2013). However, current approaches visualizing stem cells and their niche in fixed sections cannot define the true niche since the cell may still have been migrating when the tissue sample was taken. The only way to visualize cell movements in the BM with sufficient spatial/temporal resolution without physically damaging the niche is by intravital imaging of the calvaria (Lo Celso et al., 2009). While different in structure and developmental origin to the long bones, HSCs in the calvaria show identical HSC frequency and function to those found in the femur (Lassailly et al., 2013, Lo Celso et al., 2009).

Intravital imaging of mouse HSPCs in calvaria showed that by 16 hr after transplantation, the majority of cells had entered the bone, crossed the endothelium, and lodged within a few cell diameters of bone. HSPCs localized to distinct regions according to their differentiation status (Lo Celso et al., 2009); at least in the calvaria, both osteoblastic and vascular niches are not physically separate, and a cell can be located within both. However, it remains unclear whether we can extrapolate the definition of the mouse HSC niche to human. In order to study the early phases of human HSPC homing and lodgment, we adopted a similar approach used by Lo Celso et al. (2009) to track human and mouse HSPCs in the calvaria of live mice. Using time-lapse imaging, we show that both human and mouse HSCs and hematopoietic progenitor cells (HPCs) exhibit strikingly different motile behaviors. It takes human HSC-enriched cells longer than expected to find their niche at which point motility decreases. In comparison, progenitor-enriched populations continue to navigate the microenvironment. We show that blocking integrin binding within the niche can reverse the relatively non-motile phenotype of HSCs, indicating a role for integrins in the motility of HSCs after lodgment. We also found a similar pattern of motility in mouse HSPCs, where HSCs were much less motile than HPCs. Our results suggest that for human cells, the definition of lodgment should be described by their position when cells stop moving and that, on a whole-population level, migratory behavior in vivo can be used to identify a more pure population of human and mouse HSCs. Imaging of migratory behavior is crucial in order to pinpoint the location of where stem cells stop moving and lodge, which is indicative of the HSC niche. Once the location is confirmed, a more thorough analysis of the microenvironment at this location can be performed. Moreover, intravital imaging can be used to test pharmacological, antibody blocking, or genetic manipulation of molecules thought to play a role in HSC function since it allows one to determine whether functional defects occur from an inability to migrate or adhere during the distinct homing, lodgment, and mobilization processes.

## Results

### Developing Tools to Track Human/Mouse HSPCs by Intravital Microscopy

In order to visualize HSPC interactions with their niche, cells must be labeled with a fluorescent marker. Lipophilic dyes have been used to track HSPCs, but leakage of dye and contamination of microenvironment is possible (Lassailly et al., 2010). Amine reactive cell dyes do not appear to leak and are frequently used to track lymphocyte division and migration (De Clerck et al., 1994, Nolte et al., 2004). However, since CFSE and SNARF have been reported to have some negative effects on cell function, we performed analyses to find the optimal concentration (carboxyfluorescein succinimidyl ester [CFSE] 2 μM and SNARF 1.5 μM, data not shown). We confirmed non-toxicity using in vivo transplantation experiments, showing no detrimental effects on engraftment of dye-labeled HSPCs in primary and secondary recipients (Figures S1A and S1B). Furthermore, each time point and population were repeated using both dyes in order to ensure no effect on motility of a specific dye (Figure S1C). The possibility of autofluorescent cells in the BM being mistaken for dye-labeled cells is minimal since autofluorescent cells appear in both CFSE (green) and SNARF (red) detectors and appear as yellow/orange objects (Figure S1D). Any object that appears in both CFSE and SNARF channels was excluded from the analysis.

In addition to visualizing stem and progenitor populations labeled with dyes, we collected the second harmonic signal emitted by collagen in bone (cyan). The distance from the center of the cell to the closest pixel of bone surface is displayed graphically. Using this protocol, we are able to track cell position relative to the bone surface and movements over time.

### Human HSC- and HPC-Enriched Populations Take up to 4 days to Lodge in an Endosteal Niche

**Figure 1:**
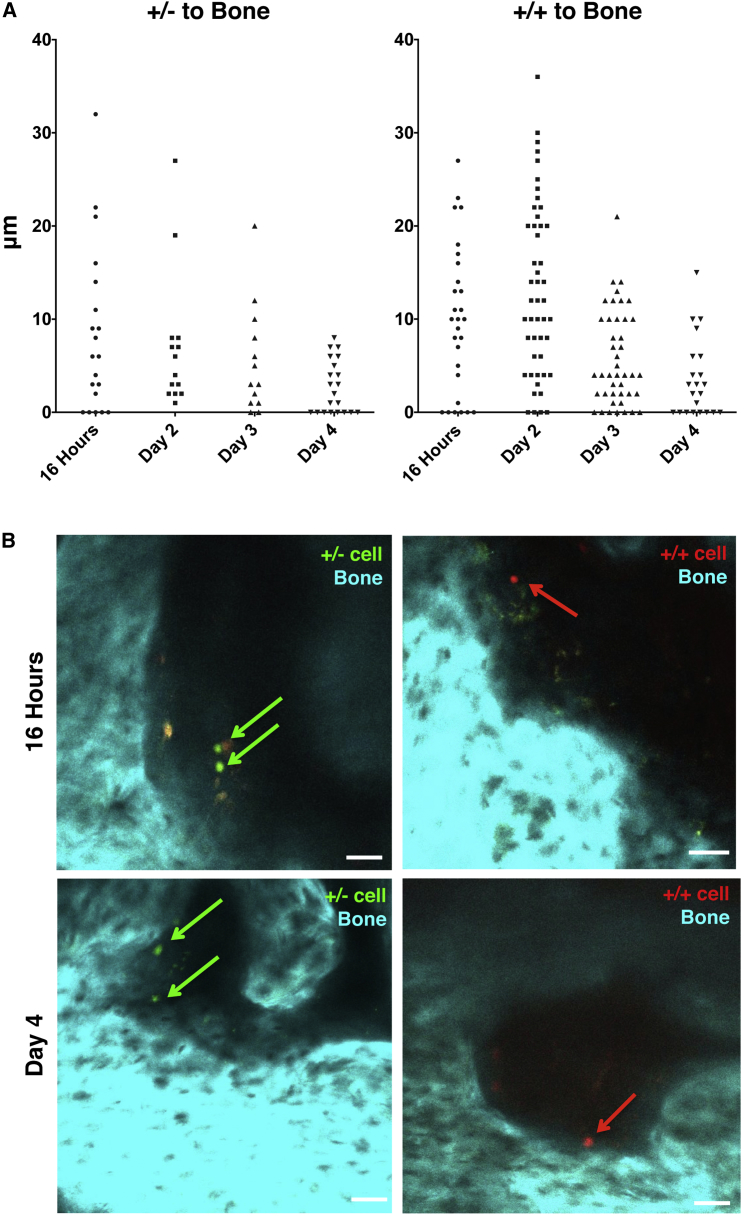
In Vivo Imaging of Human HSPCs CB mononuclear cells (CB MNCs) were flow sorted to purify Lin−CD34^+^CD38^−^ (+/−) and Lin^−^CD34^+^CD38^+^ (+/+) populations and then labeled with dyes and injected IV in NSG recipients. (A) Live distance measurements in μm of +/− and +/+ cells in the calvaria to bone at 16 hr and days 2, 3, and 4 post-transplantation. (B) Live images from calvaria of mice transplanted with +/− (green, left) and +/+ (red, right) cells at 16 hr (top) and day 4 (bottom) after transplantation. Second harmonic signal generated by the bone is cyan. Scale bars represent 50 μm. N = 9 recipients of cells from pooled CB units (two recipients for 16 hr, two recipients for day 2, three recipients of day 3, three recipients of day 4). Each recipient received cells from approximately six to nine CB donors.

A recent report from Bhatia’s group showed that human HSPCs xenotransplanted into an immunodeficient mouse resided in both vascular and endosteal regions at steady state, similar to indigenous HSPCs in human bone. The report by Bhatia’s group demonstrated functional human hematopoiesis in mice, validating the use of human-mouse BM xenografts. However, the authors did not track cell migration over time in order to describe homing and lodgment (Guezguez et al., 2013). We applied a similar xenotransplantation technique to study migration, homing, and lodgment of human HPSCs. Prior to starting these experiments, we tested our intravital imaging technique using mouse HSPCs to confirm expected results with those previously published (Lo Celso et al., 2009). Likewise, we saw that at 16 hr after transplantation mouse HSPCs had crossed the endothelium to enter the BM and were located with two to three cell diameters of the bone surface (data not shown). To track human HSPCs, we used expression of lineage markers (Lin), CD34 and CD38 to sort HSC-enriched Lin^−^CD34^+^CD38^−^ and HPC-enriched Lin^−^CD34^+^CD38^+^ cells (herein referred to as +/− and +/+). We transplanted these cells into sublethally irradiated NSG mice. By 16-hr post-transplant, the cells have crossed the endothelium and entered the BM cavity (Figure 1B, top row). However, time-lapse microscopy indicated that cells were still motile, suggesting that at 16 hr they may not be lodged in their final niche, so we continued to track cells for up to 4 days after transplantation (Figures 1A and 1B). Serial analyses showed that both +/− and +/+ populations continue to move and get progressively closer to bone (Figures 1A and 1B), eventually settling at an average distance of within one to two cell diameters (one cell diameter = 6 to 8 μm; Xie et al., 2009) of the endosteum at day 4 (Figures 1A and 1B, bottom row). It is possible that differences in position of cells over time are due to changes in niche architecture such as vascular remodeling or osteoblast loss following irradiation. As such, a more detailed analysis of transplanted cell position in relationship to other niche components after irradiation will be required. Another possible explanation for the change in position of HSPCs is that cells that are close to the endosteum persist, while those located further away are lost through apoptosis and/or have proliferated and thus lost their dyes. However, it is not currently possible to measure apoptosis of cells that reside in one location versus another in order to address this question. Furthermore, we could not detect cells that have undergone proliferation.

### HSCs that Home to the BM and Persist after Transplantation Retain Stem Cell Activity

**Figure 2:**
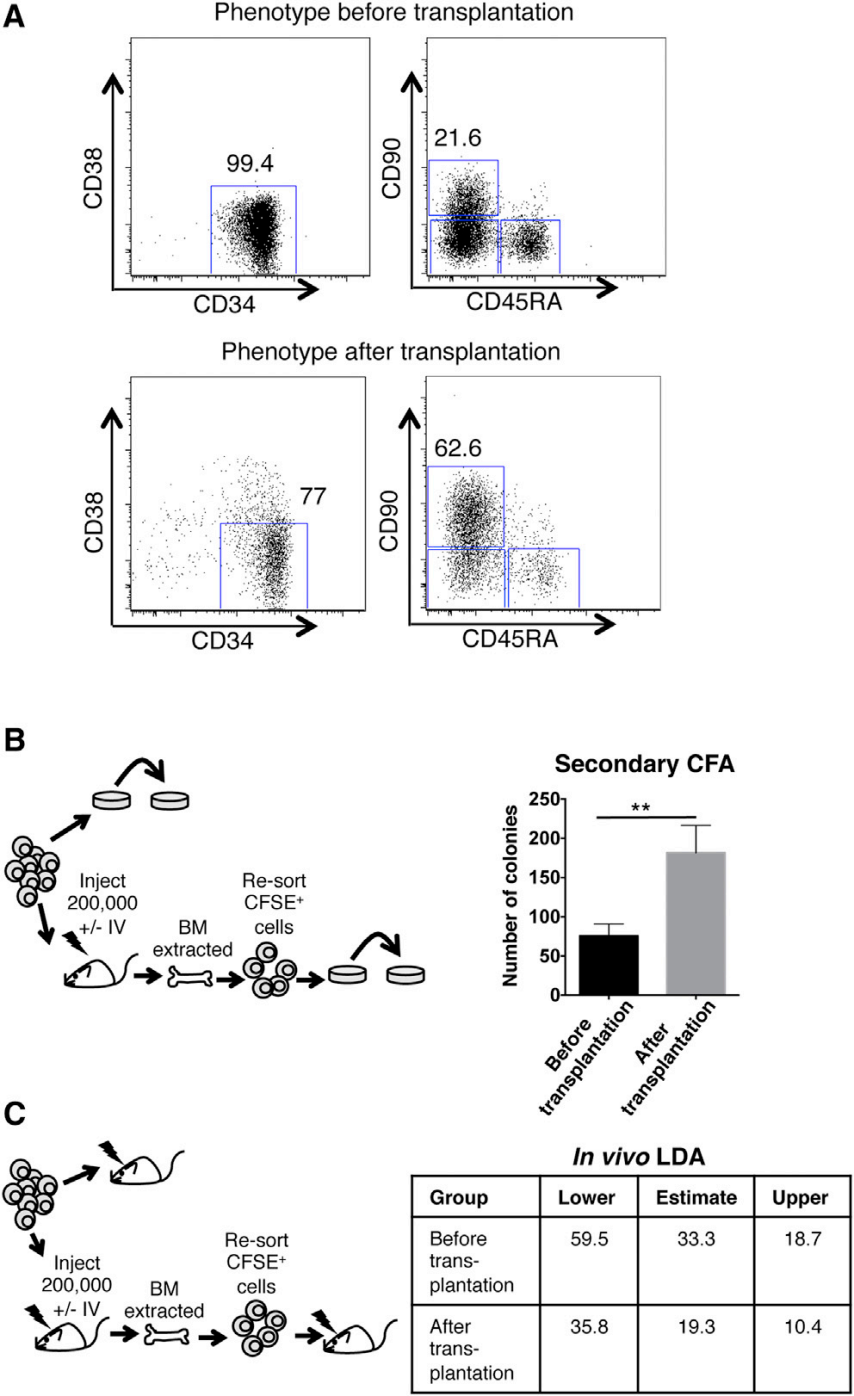
Estimation of Stem Cell Frequency of Cells that Home to and Persist in the BM after Transplantation (A) FACS analysis (top) of the CSFE^+^ CD34^+^CD38^−^ before transplantation (bottom) FACS analysis of the CSFE^+^ cells extracted from the BM of NSG mice at day 4 post-transplantation. (B) (left) Protocol to assess colony-forming ability of cells visible by microscopy; 300 Lin^−^CD34^+^CD38^−^ (+/−) cells were plated directly into methylcellulose (before transplantation group) or stained with CFSE and injected IV into NSG recipient. After homing, mice were sacrificed; BM was extracted from femurs, tibias, and iliac crests and re-sorted to obtain CFSE^+^ cells; and these were plated into methylcellulose (after transplantation group). 75,000 cells from primary colonies were obtained after 14 days and re-plated to generate secondary colonies. (Right) Number of secondary colonies. N = 5 independent experiments from pooled CB units. Error bars represent ± SD. ^∗^p < 0.05, ^∗∗^p < 0.01, ^∗∗∗^p < 0.001. (C) (left) Protocol to assess stem cell frequency after transplantation. Lineage-depleted CB MNCs were stained with Lin, CD34, and CD38 antibodies and sorted by FACS to purify Lin^−^CD34^+^CD38^−^ cells. Cells were injected intra-bone at limiting dose into sublethally irradiated recipients (before transplantation group) or stained with CFSE and injected IV into NSG recipient. After homing, mice were sacrificed, BM was extracted from femurs, tibias, and iliac crests and re-sorted by FACS to obtain CFSE^+^ cells and were injected intra-bone into the tibia at limiting dilution in sublethally irradiated recipients (after transplantation group). (Right) Engraftment at 12 weeks was assessed by flow cytometry for human CD45 multilineage (containing CD3, CD19, and CD33 cells) engraftment and scored positive if above 0.1%. ELDA software was used to calculate the frequency of repopulating cells. N = 48 mice (before and after cohort of mice used per condition: two mice each for 5, 20, and 40 cell doses, six mice each for 60 and 80 cell dose, from two independent experiments from approximately 9–12 pooled CB units).

It is possible that at 4 days after transplantation the cells that we are imaging may no longer be stem cells and have differentiated and/or lost repopulating ability. Therefore, the phenotype of transplanted cells we see may not be characteristic of stem cells. In order to determine the extent of transplanted cell differentiation, we retrieved the CFSE^+^ cells at day 4 and performed a FACS analysis of these cells. A high proportion of these CFSE^+^ cells were still CD34^+^CD38^−^, and more than 66% were CD45RA^−^CD90^+^, indicating that these CFSE^+^ cells imaged at day 4 are still phenotypically enriched in repopulating cells (Figure 2A). To confirm this functionally, we compared their secondary clonogenic capacity by colony forming unit (CFU) of the CFSE^+^ cells that had been transplanted and recovered by fluorescence-activated cell sorting (FACS) (“after transplantation”) to fresh CFSE labeled +/− cells (“before transplantation”) that were plated immediately into methylcellulose. After 2 weeks, the colonies were re-plated to generate secondary colonies (Figure 2B). The group of cells that was extracted from the mouse after transplantation showed a 2- to 3-fold increase in secondary re-plating ability (75,000 cells from “before transplantation” colonies gave 75 colonies’ 75,000 cells from “after transplantation” colonies gave 181 colonies; Figure 2B). We further performed an in vivo limiting dilution assay (LDA) experiment to assess stem cell frequency in CFSE^+^ cells before and after transplantation. Before transplantation and after transplantation cells were injected intra-bone into the tibia of sublethally irradiated NSG mice at doses ranging from 5 to 80 cells (Figure 2C, left). After 12 weeks, engraftment was assessed by flow cytometry and repopulating-cell frequency calculated. The engraftment frequency after transplantation (frequency: 1 in 19, 95% confidence interval: 1 in 10.4 to 1 in 35.8) was statistical similar to that before transplantation (frequency: 1 in 33.3, 95% confidence interval: 1 in 18.7 to 1 in 59.5) (Figure 2C, right). These analyses showed that CFSE^+^ cells imaged at day 4 were still functionally enriched in cells with repopulating and multipotential activity, suggesting that we are still imaging bona fide stem-enriched cells.

### HSC- and HPC-Enriched Populations Exhibit Distinct Motility Behaviors

**Figure 3:**
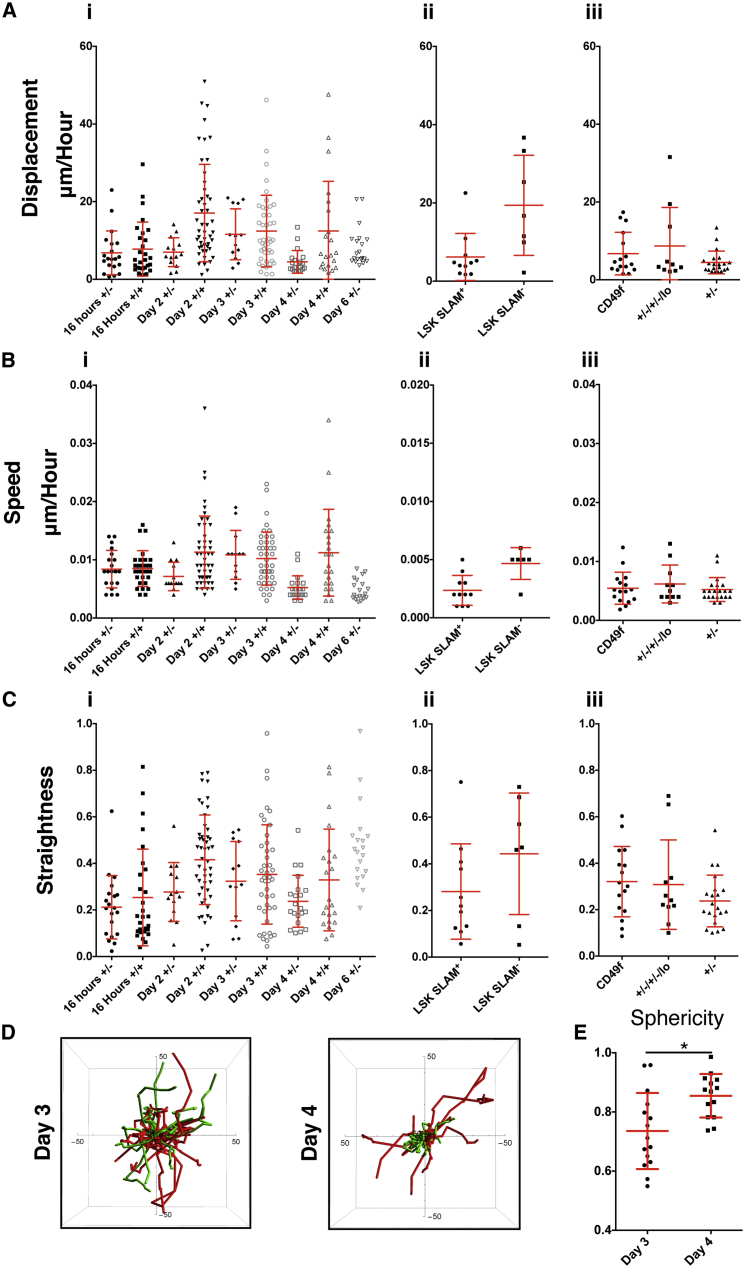
In Vivo Migration of Human and Mouse HSPCs CB MNCs were lineage depleted, stained with antibodies, and flow sorted to purify Lin^−^CD34^+^CD38^−^ (+/−) and Lin^−^CD34^+^CD38^+^ (+/+) populations. Cells were labeled and injected IV into NSG recipients. (A–C) The displacement per hour (A), speed (B), and straightness (C) of (i) +/− and +/+ cells in μm in the calvaria at 16 hr and at days 2, 3, 4, and 6 post-transplantation. N = 10 recipients of cells from pooled CB units (two recipients for 16 hr, two recipients for day 2, three recipients of day 3, three recipients of day 4, and one recipient of day 6). Each recipient received cells from approximately six to nine CB donors. (ii) BM obtained from femurs and tibias of C57Bl/6 mice were lineage depleted, stained with Lin, SCA1, cKIT, CD150, and CD48 antibodies, and flow sorted to obtain LSK SLAM^+^ (Lin^−^, SCA1^+^, cKIT^+^, CD48^−^, CD150^+^) and LSK SLAM^−^ (Lin^−^, SCA1^+^, cKIT^+^, not CD48^−^, CD150+). The cells were labeled and injected IV into NSG recipients. The displacement per hour (A), speed (B), and straightness (C) of LSK SLAM^+^ and LSK SLAM^−^ in the calvaria at 16 hr is shown. N = 2 recipients at each time point of cells from pooled BM from approximately 10 C57Bl/6 donor mice for each recipient. (iii) CB MNCs were lineage depleted; stained with CD38, CD34, CD45RA, CD90, and CD49f antibodies (“CD49f”) or the cell dye Rhodamine123 (+/−/+/−/lo); and flow sorted to purify HSCs compared with HSPCs (CD34^+^/CD38^−^). Cells were labeled and injected IV into NSG recipients. Displacement per hour (A), speed (B), and straightness (C) of cells in the calvaria at 4 days after transplantation is shown. N = 3 recipients of CD49f^+^ cells, N = 2 recipients of +/−/+/−/lo cells, and N = 3 recipients of +/− cells of cells from approximately six to nine pooled CB units for each recipient. The +/− data in this figure are the same data from Figure 3A. (D) The actual migration tracks for all +/− (green) and +/+ cells (red) from Figure 3A were overlaid on a 3D track plot (Wolfram Mathmatica) for day 3 (left) and day 4 (right). Total track length is displayed which ranges from 1–2 hr per cell. (E) Sphericity for +/− cells at day 3 and day 4. Data obtained from the same cells in Figure 3A.

Since HSCs appeared to keep moving after transplantation, we performed live cell tracking to determine whether and/or when the cells stop migrating and whether there are any dynamic differences during their homing. At 16 hr, days 2, 3, 4, and 6 after transplantation, we tracked cells for 2 to 4 hr and calculated the displacement per hour, speed, and straightness. Displacement per hour describes the distance between the start and end point of migration corrected for time, which shows overall movement of the cell. Straightness is a measure of whether a cell is moving in a straight line (directed motion) or moving randomly; a value closer to 1 equals a straight path, while a lower value is indicative of random walk. Displacement values showed that 16 hr after transplantation, there were no differences between +/− and +/+ populations (Figure 3Ai). Both populations have an elongated morphology and are highly motile by day 3 (Figure 3Ai; Movie S1). However, by day 4 after transplantation, +/− cells dramatically decrease their displacement and remain rounded while the majority of +/+ cells remain highly motile and elongated (Figure 3Ai; Movie S2), and although there is a smaller subpopulation that are still moving, the majority of +/− cells stay within a few microns of their starting position. Statistical analysis by ANOVA test showed a significant difference in displacement between +/+ and +/− cells (p = 0.001) and between time points (p = 10^−06^) (comparing days 3 and 4). The difference in motility is highlighted when migration tracks are displayed graphically; the tracks of +/− (green) and +/+ (red) cells show similar patterns at day 3 (Figures 3D, left, and S2A and S2B), while the same analyses show a marked difference in pattern at day 4 (Figures 3D, right, and S2A and S2B). Analysis of cell speed showed a similar pattern whereby both had a similar speed at day 3, but at day 4, +/− cells had a lower speed than +/+ cells. Statistical analysis by ANOVA test showed a significant difference in speed between +/+ and +/− cells (p = 10^−05^) and between time points (p = 10^−09^) (comparing days 3 and 4) (Figure 3Bi). The pattern of migration was also changed significantly over days 3 and 4, with +/− cells exhibiting a more random walk type of motility by day 4, as indicated by lower straightness values (Figure 3Ci) (p = 10^−05^).

Observations of cell movements indicated that +/− cells changed their morphology between days 3 and 4 after transplantation. In order to determine whether there was a significant difference in cell shape, we measured the sphericity of HSPCs (+/− cells) when they were at their most motile on Day 3 and at Day 4, when the majority of cells stopped moving. These analyses showed that +/− cells at day 3 are statistically less spherical (a value closer to 0) and thus more polarized compared with +/− cells at day 4, which were more spherical (a value closer to 1; Figure 3E). Together, the change in morphology combined with a difference in motility, as measured by displacement, speed, and straightness, indicates a change in behavior of the majority of the +/− population over time.

To determine whether the distinct pattern of +/− motility persists beyond 4 days, we imaged 2 days later at day 6. Hardly any +/+ cells were observed at this later time point, probably due to high proliferation of the cells and dilution of the dye, or apoptosis, and are not included in the data. On a whole population level, the +/− population was more motile at day 6 than day 4 (Figure 3Ai). However, on closer inspection, it appears that there are two distinct subpopulations; one that remains non-motile and now a larger population of motile cells. Indeed, further analysis of speed and straightness parameters showed that there was no difference in speed between days 4 and 6 (Figure 3Bi), but an increase in straightness, whereby cells were exhibiting a more directed motion type of movement (Figure 3Ci). In order to determine whether differences between HSC and HPC motility were specific to human HSPCs, we also tracked the migration of mouse HSPCs. We FACS-sorted mouse Lin^−^SCA1^+^cKIT^+^CD48^−^CD150^+^ (LSK SLAM^+^, HSC) and the rest of the Lin^−^SCA1^+^cKIT^+^ (LSK) cells that were not CD150^+^CD48^−^ (this include HPC1, HPC2, and MPPs following Sean Morrison’s group definition; Oguro et al., 2013). For simplicity, herein we call these cells (LSK SLAM^−^, HPC). Because of high HPC proliferation, we could only track LSK SLAM^−^ cells for 16 hr. Similar to human HSPCs, mouse HSCs exhibited a reduced displacement and had a rounded morphology (Figure 3Aii; Movie S3) compared with their HPC counterparts, which were highly motile and elongated (Figure 3Aii; Movie S4). Analysis of speed indicated a significant difference between LSK SLAM^+^ and LSK SLAM^−^ cells (Figure 3Bii; Movies S3 and S4), and LSK SLAM^−^ cells exhibited a trend, but not significant difference, toward more directed motion pattern of motility as measured by straightness (Figure 3Cii; Movies S3 and S4), indicating that the differences between stem and progenitor cell motility is consistent between species and not specific to human cells in the xenotransplantation setting.

### Heterogeneity in Motile Behavior Is Not Accounted for by Stem Cell Purity

As indicated in Figure 3Ai, some heterogeneity exists in the behavior of the +/− stem cell-enriched population where not all the +/− cells stop migrating at day 4. We hypothesized that more highly purified stem cells would be the least displacing, while those still highly migratory were of a more differentiated population. To test this, we sorted highly purified HSC populations based on expression of Lin^−^, CD34^+^, CD38^−^, CD90^+^, CD45RA^−^, CD49f^+^ (Notta et al., 2011) (herein called CD49f^+^) and CD34^+^, CD38^−^, CD90^+^, CD45RA^−^, and efflux of Rhodamine 123 (Kim et al., 1998) (herein called +/− /+/−/lo). We labeled and transplanted these cells and imaged 4 days later. There were no differences in location between +/−, CD49f^+^, and +/−/+/−/lo populations (Figure S2C). Surprisingly, however, the displacement, speed, and straightness were also similar to +/− cells (Figures 3Aiii, 3Biii, and 3Ciii), and there was still heterogeneity in the motility of cells. This could be due to further heterogeneity in the CD49f^+^ and +/−/+/−/lo phenotypes and thus functional differences that result in a difference in migration. Alternatively, it is possible the CD49f^+^ and +/−/+/−/lo populations are fairly pure and, depending on the cues that the cell receives from the environment following transplantation, have different dynamic properties. In order to further clarify why there is heterogeneity in terms of behavior within even the highly enriched HSC population, it would be necessary to assess the cell-cycle status or gene expression profile of motile versus non-motile cells; however, it is not currently possible to purify the two populations from the BM based on motility. Moreover, even though the dyes used to track cell divisions are routinely used to track cell divisions by flow cytometry, due to differential scattering of reflected light dependent on how deep in the BM the dye-labeled cell is positioned, unfortunately, we could not use the dye to count cell divisions by imaging and thus correlate position with proliferation status.

### Could Conditioning Regimes Account for the Differences in HSPC Motility Observed?

**Figure 4:**
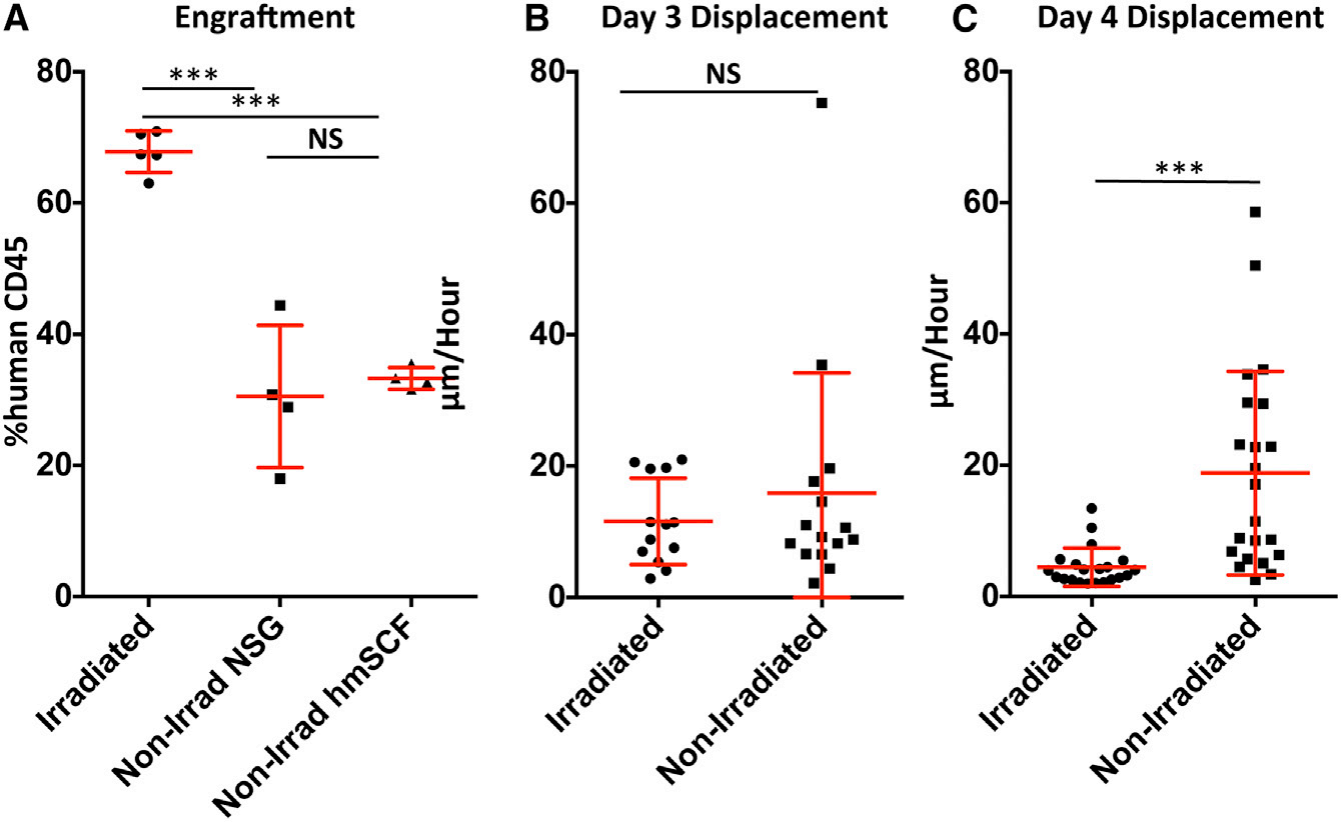
Role of Conditioning Regimes in the Motility Behavior of HSPCs (A) 100,000 lineage-depleted CB MNCs were IV injected into sublethally irradiated NSG (3.75 Gy 24 hr prior to transplantation), non-irradiated NSG, and non-irradiated hmSCF NSG mice. At 12 weeks after transplantation, BM from tibia, femur, and iliac crests was obtained and stained with human CD45 antibody and analyzed by flow cytometry. Error bars represent ± SD. ^∗∗∗^p < 0.001. N = 5 irradiated NSG, N = 4 non-irradiated NSG, and N = 4 non-irradiated hmSCF-NSG recipients of pooled CB units. (B and C) CFSE-labeled FACS-sorted Lin^−^CD34^+^CD38^−^ cells were transplanted IV into 3.75 Gy sub-lethally irradiated NSG (3.75 Gy 24 hr prior to transplantation), non-irradiated NSG, or hmSCF NSG mice. Data indicate displacement per hour of +/− cells in μm in calvaria at 3 (B) and 4 (C) days after transplantation. Error bars represent ± SD. ^∗∗∗^p < 0.001. N = 3 irradiated mice, N = 4 non-irradiated mice.

Since sublethal irradiation was used to condition the mice for transplantation, it is possible that the difference in motility of HSPCs could be due to changes in the microenvironment and/or in the hematopoietic cells after conditioning. To investigate this possibility, we performed identical experiments into non-irradiated NSG and non-irradiated NSG human membrane bound stem cell factor (hmSCF) mice. NSG-hmSCF mice were recently reported as an improvement to the NSG model, as these mice do not require irradiation to permit engraftment of human hematopoietic cells (Brehm et al., 2012, Takagi et al., 2012). In our hands, we found a significant reduction in engraftment in non-irradiated NSG and NSG-hmSCF recipients compared with irradiated mice; however, there were no differences in engraftment between non-irradiated NSG and non-irradiated NSG-hmSCF recipients (Figure 4A); therefore, the two non-irradiated groups were merged in further analyses. We performed motility comparisons at 3 versus 4 days post-transplantation. At 3 days after transplantation, there was no difference in the average displacement between of HSPCs between non-irradiated versus irradiated mice (Figure 4B). At day 4 post-transplantation, HSPCs were more motile in non-conditioned NSG and NSG-hmSCF compared with irradiated mice (Figure 4C). However, looking more closely at the displacement values from non-irradiated recipients at day 4 (Figure 4C), it appears that there are two subpopulations with around half of the cells having a similar low displacement to irradiated mice and the remaining cells being motile. This is significant since there is around a 50% reduction in engraftment in non-irradiated recipients (Figure 4A). We suggest that fewer available niches in non-irradiated mice might explain these results. Thus, around half of the cells stop moving since they have found an empty niche and are able to contribute to engraftment, while the remaining cells are unable to find an empty niche and keep moving and do not receive the cues necessary to survive and therefore do not engraft. We nevertheless could not exclude that some microenvironmental changes occur between days 3 and 4 in conditioned mice, which are responsible for this change in motility of HPCs. Further analysis using new mouse models not requiring conditioning to achieve comparable engraftment levels will definitively address whether the increase in available niches correlates with an increase in less motile HSCs (similarly to conditioning), as in these mice more niche space is available for transplanted HSPCs due to functional impairment of endogenous HSPCs (Cosgun et al., 2014).

In order to determine whether there are any gene changes in transplanted +/− cells between 3 and 4 days after transplantation that would account for a decrease in motility, for example, by increased adhesion, we performed microarray analysis on +/− and +/+ cells that had been CFSE labeled and transplanted into sublethally irradiated NSG mice and recovered them from the BM by FACS sorting at 3 and 4 days after transplantation. However, we did not detect any upregulation of genes mediating adhesion such as integrins and cadherins at day 4 that would explain why +/− cells stop moving (data not shown). This is likely because the function of adhesion molecules is not regulated at the gene transcription level and/or that adhesion molecule function is stable after transplantation and HSCs keep moving until they find an empty niche that expresses the correct receptor(s).

### Disruption of Integrin Binding within the Niche 4 Days after Transplantation Reverses the HSPC-Specific Reduction in Motility 4 Days after Transplantation

**Figure 5:**
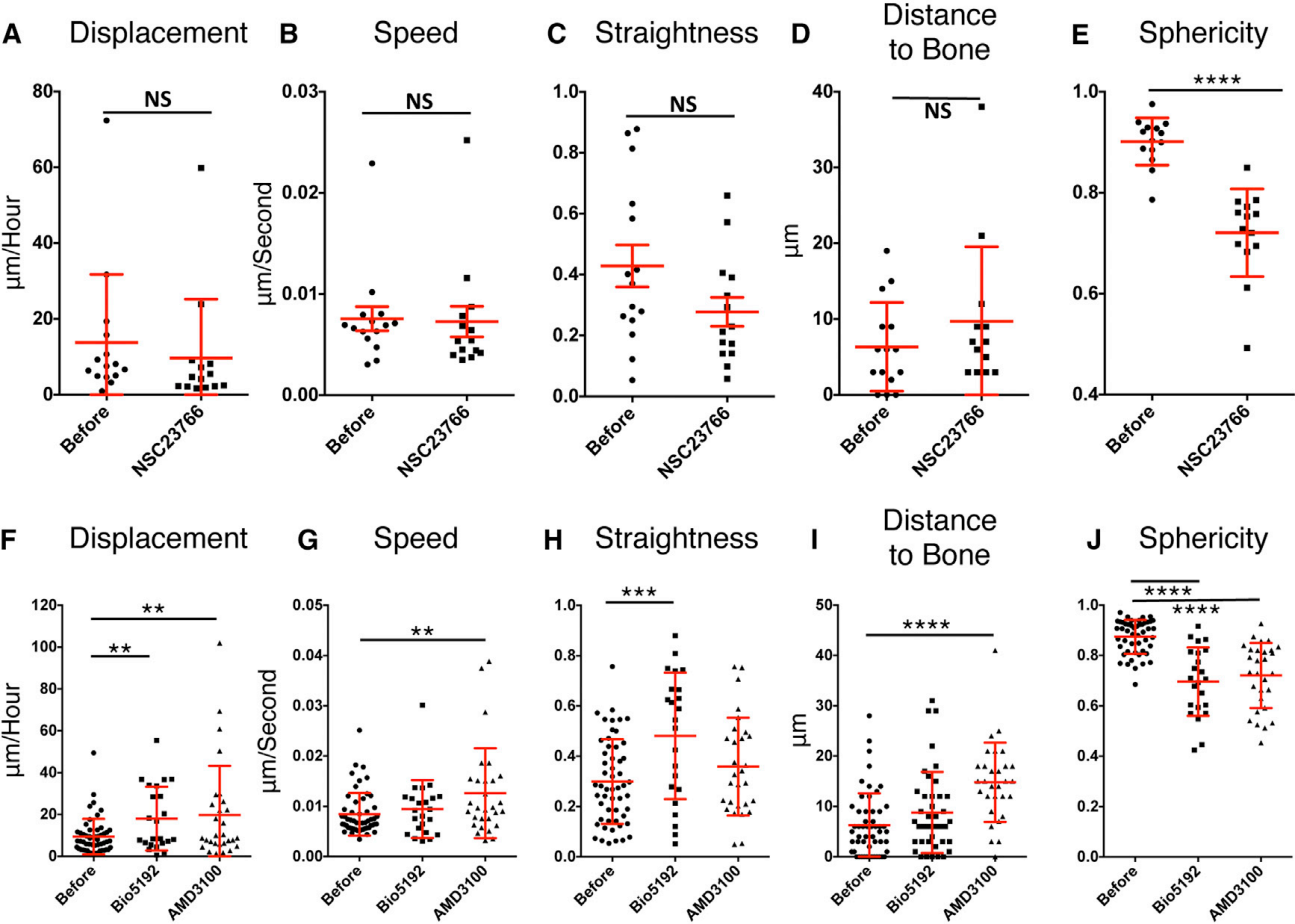
In Vivo Blocking of Integrins Reverses the Non-motile Phenotype of HSPCs (A–E) In vivo treatment at day 4 post-transplantation of NSG mice with RAC1-GEF inhibitor NSC 23776 (3 mg/kg). Displacement (A), Speed (B), Straightness, (C) Distance to Bone, (D) and Sphericity (E) were measured from cells imaged in the calvaria, calculated before and after treatment. N = 2 recipients of six to nine pooled CB units for each recipient. (F–J) In vivo treatment at day 4 post-transplantation of NSG mice with beta1 inhibitor-Bio 5192 (1 mg/kg) or CXCR4 antagonist-AMD3100 (1mg/kg). Displacement (F), speed (G), straightness (H), distance to bone (I), and sphericity (J) were measured from cells imaged in the calvaria and were calculated before and after treatment. N = 2 recipients (AMD3100) and 3 recipients (Bio 5192) of six to nine pooled CB units for each recipient. Error bars represent ± SD. ^∗∗^p < 0.01, ^∗∗∗^ p< 0.001; ^∗∗∗∗^p < 0.0001.

When studying the movies of HSPCs migrating (Movies S1 and S2), we saw that while moving HSPCs continually changed shape but when stopped they remained rounded. In order for a cell to migrate it must polarize, which leads to changes in cell shape. We wondered whether +/− cells became less motile at day 4 after transplantation due to changes in their ability to polarize. One of the key players in cell polarization is *Rac1*. It was shown that deletion of *Rac1* in HSPCs inhibited their ability to form cobblestones in vitro and the frequency of *Rac1*^−/−^ HSPCs that were able to home to and be retained in vivo at the endosteal surface was reduced (Cancelas et al., 2005). In order to determine whether altering RAC1-mediated polarization could change the motility of +/− cells at day 4, we used a RAC1-GEF specific inhibitor (NSC23766) in vivo. To do this, +/− cells were imaged 4 days after transplantation to confirm the expected pattern of +/− immobility. Afterward, NSC23766 was injected and imaging was continued for up to 4-hr post-injection. Surprisingly, there was no increase in displacement (Figure 5A), speed (Figure 5B), or straightness (Figure 5C) of +/− cells, and their position relative to the bone surface did not change (Figure 5D). However, there was a highly significant decrease in sphericity indicating that RAC1 treatment does affect polarization of +/− cells (Figure 5E). Thus, it appears that inhibiting polarization is not sufficient to change migration, and there are other molecules that affect motility, for instance adhesion molecules retain, or anchor, HSPCs in their niche.

One of the most extensively characterized signaling pathways in HSC homing is the CXCR4/SDF1 axis. CXCL12 binding to CXCR4 enhances the affinity of integrins, in particular VLA-4, which mediates HSC cell adhesion in the niche (Hartmann et al., 2008, Peled et al., 1999a, Peled et al., 2000). Impairment of integrin or CXCR4/SDF1 expression or neutralization of their function by antibody treatment is known to be associated with reduced HSC homing (Kollet et al., 2001, Peled et al., 1999b, Qian et al., 2006, Wagers et al., 2002). Treatment with the CXCR4 receptor antagonist, AMD3100, is a potent mobilizing agent (Broxmeyer et al., 2005, Liles et al., 2003), and inhibition of VLA-4 (via Bio5192, a small molecule inhibitor) alone and in combination with CXCR4 antagonists was shown to mobilize long-term HSPCs to the periphery more effectively than with AMD3100 alone (Bonig et al., 2009, Ramirez et al., 2009, Leone et al., 2003). However, the precise role of integrin(s) in the processes of extravasation from blood vessels into the BM niche versus their function in migration and lodgment in the niche is difficult to separate. In order to examine the role of VLA-4 specifically in the migration and lodgment of HSCs at day 4, we transplanted CFSE labeled cells in vivo. At 4 days later, cells were imaged to confirm the expected lack of migration of +/− cells and continued movement of +/+ cells. We then injected VLA-4 inhibitor (Bio 5192) and continued to track migration up to 4 hr after injection. Addition of Bio 5192 resulted in a significant increase in displacement (Figure 5F; Movie S5) and straightness (Figure 5H; Movie S5), a trend of, although not significant, increased distance to the bone surface (Figure 5I) and a significant decrease in sphericity (Figure 5J), indicating a role for VLA-4 in the non-motile phenotype of stem cells 4 days after transplantation. However, there was a subpopulation of cells that did not respond to this inhibition and remained relatively non-motile; thus, it is likely that other integrins also regulate HSPC adhesion in the niche. Since CXCR4/SDF1 might regulate the affinity of various integrins in addition to VLA-4, we treated cells with the CXCR4 antagonist, AMD3100. Similar to beta1 inhibition, AMD3100 treatment significantly increased the displacement (Figure 5F; Movie S6), speed (Figure 5G; Movie S6) and trend, but not significant, increase in straightness (Figure 5H; Movie S6) of the majority of +/− cells, their distance from the bone surface (Figure 5I), and significantly decreased sphericity of the cells (Figure 5J), although again there was still a subpopulation of cells that did not respond to treatment. It is likely that adhesion and retention in the niche are controlled by a complex array of adhesion molecules, extracellular matrix components, and that retention to specific stromal cell subtypes involves different molecules that are yet to be defined.

## Discussion

The location and composition of the HSC niche are areas of intense debate in the stem cell field with numerous studies describing the niche in the mouse (Ellis et al., 2011, Kiel et al., 2005, Nombela-Arrieta et al., 2013, Zhang et al., 2003, Sugiyama et al., 2006, Méndez-Ferrer et al., 2010, Calvi et al., 2003), human trephine sections (Guezguez et al., 2013), and zebrafish (Tamplin et al., 2015). However, although a recent publication described changes in motility of HSPC in the context of parasitic infection (Rashidi et al., 2014), there is very little published information regarding the dynamics of HSPC migration in vivo and how this correlates with functionality. Dr. Nilsson’s group reported that just 5 hr after mouse HSC transplantation, these cells can be further enriched functionally by extracting them from the endosteal region compared to the central bone region (Grassinger et al., 2010), demonstrating that phenotypically identical HSCs isolated from different regions of the BM had different biological potential. Our data add further information on the dynamic behavior of HSPCs by tracking human and mouse HSC and HPC populations in vivo and showing that there are significant differences in their motility. Specifically, we saw that HSCs exhibit a change in motility after transplantation to become less motile, whereas most HPCs remain motile. This could be due to a cell-intrinsic effect where cells are programmed to be less motile or due to cell extrinsic cues from the microenvironment that direct the cells to stop migrating. The probability of an HSC encountering the correct extrinsic signal increases over time, which may account for the length of time for the population as a whole to stop moving and why there are some non-motile cells at earlier time points. The motility data obtained 3 days after transplantation indicate that HSCs are capable of displacing equally as well as HPCs. Therefore, the latter suggestion is more likely, and once an HSC has found an empty niche that expresses the correct receptor/ligand pair, it adheres and is anchored at that position. Pharmacological blocking with inhibitors of VLA-4 and CXCR4 reversed the non-motile phenotype of +/− cells, indicating that the anchor may be VLA-4, and other, integrins dependent. Interestingly, our results demonstrate that the dynamic behaviors that distinguish HSCs from HPCs are consistent in both mouse and human, indicating that motility behavior may be a way to discriminate between stem and progenitor cells. The differences in motility between stem and progenitor populations could be explained by differences in niche requirements between stem and progenitor cells; stem cells are dependent on the niche for survival, quiescence, and proliferation, while progenitors do not require the same niche interactions, if any, for their function.

Conventionally, the location of transplanted cells 16 hr after transplantation is used to describe the homing niche for HSPCs (Lapidot et al., 2005). However, the data presented here suggest that the position of HSCs at 16 hr after transplantation cannot be used to define the niche since cells are still migrating. Therefore, we suggest that lodgment of human HSCs be defined as their position when they stop moving. The only method to determine when a cell has stopped migrating is by intravital imaging of live, intact BM. At present, this is only possible in the calvaria. As previously reported, mouse HSCs in the skull and femurs are present at similar frequencies and are functionally identical (Lassailly et al., 2013), indicating the presence of a functional HSC niche in the calvaria. Since HSCs have not yet been imaged live in the long bones without first damaging the niche architecture (Köhler et al., 2009, Lewandowski et al., 2010, Xie et al., 2009) or at high resolution (Branchini et al., 2010) the calvaria represents the only BM cavity, which can be imaged non-invasively and with sufficient resolution to track single-cell migration and proliferation at this time. As indicated in our time-lapse imaging, HSCs continue to migrate after transplantation, and as such, it is difficult to pinpoint the location of the stem cell niche. The data show that one needs to answer questions regarding HSC homing and lodgment using multiple approaches, including live tracking analyses to determine when stem cells stop moving and the position of the cells to define where in the niche they lodge. Observing the effect on location and motility after targeting specific stromal cell populations and/or niche-hematopoietic signaling pathways should help further characterize the human HSC niche.

## Experimental Procedures

### Labeling, Transplantation, and Imaging of HSC/HPCs for Tracking

Human HSPCs were obtained from cord blood (CB) and mouse HSPCs obtained from C57BL/6 BM. For further details on human and mouse HSPC purification, labeling, and transplantation, please see Supplemental Experimental Procedures.

### RAC1, Beta1 Inhibition, and CXCR4 Antagonism of HSC Motility

To determine the effect of inhibitors on cell migration, +/− cells were transplanted into sublethally irradiated NSG recipients. At 4 days after transplantation, cells were imaged by intravital microscopy. After 2 to 3 hr of imaging, NSC 23766 (3 mg/kg, Sigma), Bio 5192 (1 mg/kg, Tocris), or AMD3100 (1 mg/kg, Sigma) were injected IV into the mouse, and imaging was resumed 30 min later for up to 4-hr post-injection.

### Image Analysis and Cell Tracking

Each z stack of images is rendered in 3D using Imaris software (Bitplane). To correct for drift or breathing artifacts, a spot is defined that does not move, and all time points are corrected to this spot. An iso-surface is generated for bone, and spot function is used to find a cell(s) of interest. The distance between center of the cell and closest pixel of bone surface is calculated using Imaris XT extension (Bitplane), and results are entered into Prism (GraphPad Software). The mean is highlighted with a red horizontal line, and error bars represent ± SD. The Shapiro-Wilk test was performed to assess normality of raw and log-transformed data. Two-way ANOVA with interaction term was performed to assess differences between groupings where two variables (phenotype and time point) exist. In experiments that compared one variable, t test was used to compare differences. Statistical analyses were performed using R 3.0.2.

To calculate displacement, the distance between the start and end point of each migration track in a straight line was calculated. Each track was corrected by time to give displacement per hour. Displacement in a straight line from start and end points was used in preference to track length because a cell moving randomly with no overall movement can have the same track length as a cell moving over a larger distance in a straight line. In-built algorithms generate speed and straightness measurements. Straightness describes whether the cell moves in a straight line (a value close to 1) or in a random direction (a value close to 0). Actual migration tracks were generated by the “Tube Plot” algorithm in MatLab (Wolfram Mathmatica).

Sphericity was calculated by measuring the area and perimeter of a single section through the center of a cell using this equation:

$$\text{x}=\left(4\times \text{Area}\times \pi \right)/\left({\text{Perimeter}}^{\wedge }2\right).$$

Further information can be found in Supplemental Experimental Procedures.

## Supplemental Information

Document S1. Supplemental Experimental Procedures and Figures S1 and S2

Movie S1. A Single Z Section Showing Human +/−, which Is Green, and +/+, which Is Red, Cell Migration at Day 3 after Transplantation Endothelial cells on blood vessels are shown in magenta. Scale bar represents 40 μm.

Movie S2. A Single Z Section Showing Human +/−, which Is Green, and +/+, which Is Red, Cell Migration at Day 4 after Transplantation Endothelial Cells on Blood Vessels Are Shown in Magenta, and the Bone Surface in Cyan. Scale bar represents 50 μm.

Movie S3. A Single Z Section Showing a Mouse SLAM+ Cell, in Green, Migrating at 16 hr after Transplantation Endothelial cells on blood vessels are shown in magenta, and the bone surface is in cyan. Scale bar represents 50 μm.

Movie S4. A Single Z Section Showing a Mouse LSK+ Cell, in Red, Migrating at 16 hr after Transplantation Endothelial cells on blood vessels are shown in magenta, and the bone surface is in cyan. Scale bar represents 40 μm.

Movie S5. A Single Z Section Showing Human +/− Cells in Green 4 Days after Transplantation and IV Injection of Bio5192 The bone surface is shown in cyan and autofluorescence in orange. Scale bar represents 40 μm.

Movie S6. A Single Z Section Showing Human +/− Cells 4 Days after Transplantation and IV Injection of AMD3100 Endothelial cells are shown in magenta. Scale bar represents 40 μm.

Document S2. Article plus Supplemental Information
