# Supplementary material for: Different Motile Behaviors of Human Hematopoietic Stem versus Progenitor Cells at the Osteoblastic Niche
Source: Stem Cell Reports. 2015 Oct 8;5(5):690–701. doi: 10.1016/j.stemcr.2015.09.003 (PMC4649139; doi:10.1016/j.stemcr.2015.09.003)
Supplement: Document S1. Supplemental Experimental Procedures and Figures S1 and S2 [file mmc1.pdf]

Stem Cell Reports, Volume *5*

Supplemental Information

Different Motile Behaviors of Human Hematopoietic Stem versus Progenitor Cells at the Osteoblastic Niche

Katie Foster, François Lassailly, Fernando Anjos-Afonso, Erin Currie, Kevin Rouault-Pierre, and Dominique Bonnet

Supplemental Figure 1

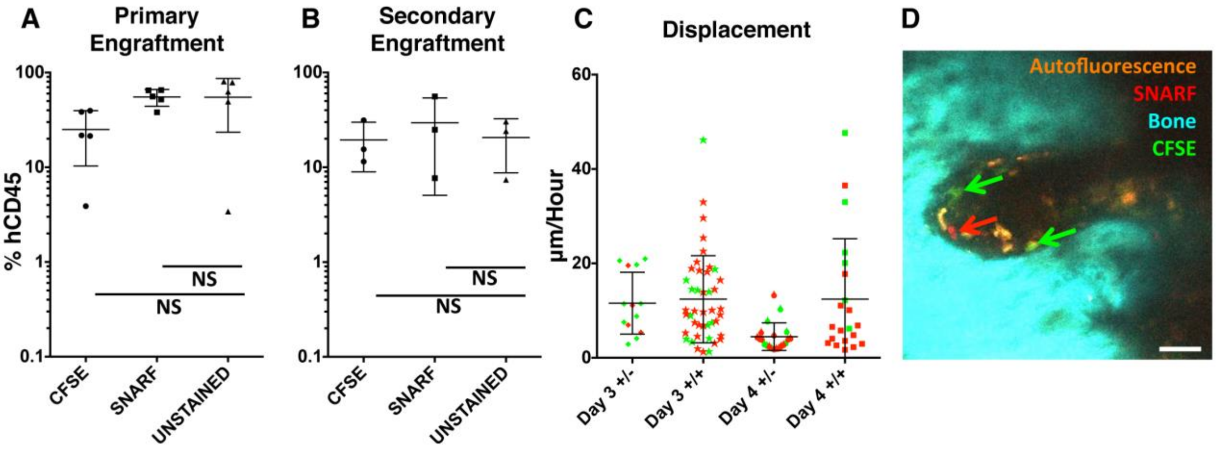

Supplemental Figure 1 Legend:

Cell labeling and Image analysis protocol, related to Figure 1. (A & B) Umbilical cord blood mononuclear cells were lineage depleted, and 100,000 Lin^- cells were injected IV into the tail vein of 3.75Gy sub-lethally irradiated NSG recipients, N = 5 CFSE recipients, N = 5 SNARF recipients and N = 5 unstained recipients. After 12 weeks, BM was extracted and an aliquot analyzed by flow cytometry to measure the engraftment of human cells (% Human $CD45^+$). The remaining cells from the 5 recipients per group were pooled and depleted for mouse CD45 cells and transplanted by IV injection into the tail vein of three secondary 3.75Gy sub-lethally irradiated NSG mice, N = 3 CFSE recipients, N = 3 SNARF recipients and N = 3 unstained recipients. Error bars represent +/- SD *p < 0.05, **p < 0.01, ***p < 0.001. (C) Displacement per hour of +/+ and +/- cells at day 3 and 4 taken from Figure 3Ai. Cells that were stained with CFSE are colored in green and cells that were labeled with SNARF are shown in red. (D) Representative image of CFSE and SNARF labeled cells in the calvaria to show specificity of each dye (only present in the green or red channel, see arrows) and auto-fluorescence, which appears in both channels and thus is colored yellow/orange. Objects, which appear in both channels, are excluded from further analysis. Scale bar = 50um.

Supplemental Figure 2

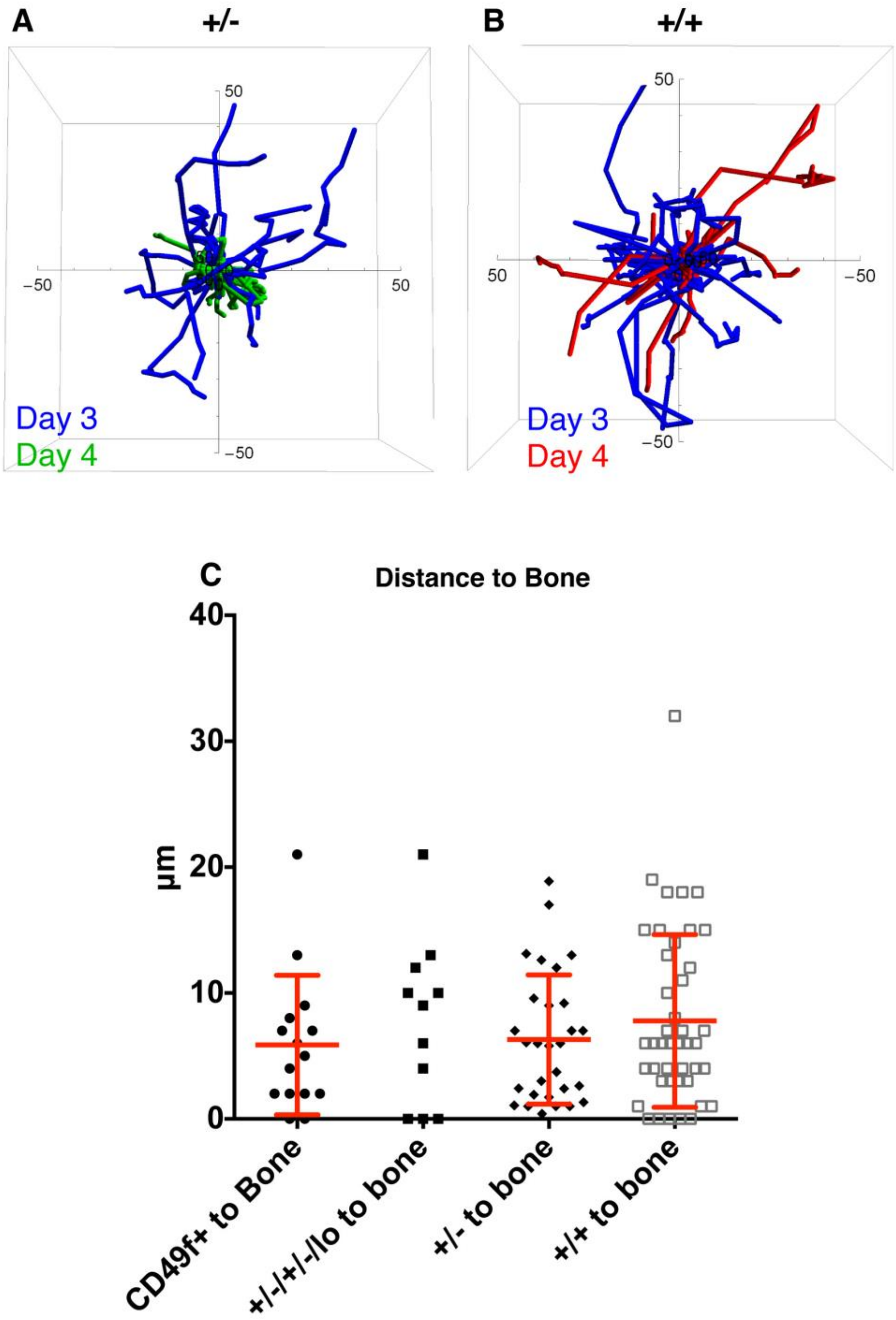

Supplemental Figure 2 Legend:

Extended *in vivo* tracking plots of human HSPCs (related to Figure 3). Umbilical cord blood mononuclear cells were lineage depleted, stained with antibodies recognizing Lin, CD38 and CD34 and flow sorted to purify $Lin^-CD34^+CD38^-$ (+/-) and $Lin^-CD34^+CD38^+$ (+/+) populations. The cells were labeled with cell dyes and injected into 3.75Gy sub lethally irradiated NSG recipients. (A) The track plot of +/- cells at day 3 (blue) and day 4 (green) are overlaid to show the difference in motility between the two time points. This is the same data as shown in Figure 3D with Day 3 and 4 tracks shown on the same plot rather than +/- and +/+ tracks on the same plot. Total track length of cells in Figure 3A, which varies from 1-2 hours in length. (B) Track plots of +/+ cells at day 3 (blue) and day 4 (red) are overlaid to show a similar pattern of motility at the two time points. This is the same data as shown in Figure 3D with Day 3 and 4 tracks shown on the same plot rather than +/- and +/+ tracks on the same plot. Total track length of cells in Figure 3A, which varies from 1-2 hours in length. (C) Umbilical cord blood mononuclear cells were lineage depleted, stained with antibodies recognizing CD38, CD34, CD45RA, CD90 and CD49f or the cell dye Rhodamine123 and flow sorted to purify HSCs stained with CFSE and transplanted into sub-lethally irradiated NSG mice. Four days after transplantation, the distance between CFSE labeled $CD49f^+$, +/-/+/-/lo, +/+ and +/- cells and both bone surface was calculated. N = 3 recipients of $CD49f^+$ cells, N = 2 recipients of +/-/+/-/lo cells and N = 3 recipients of +/- and +/+ cells from approximately 6-9 pooled CB units for each recipient. Error bars represent +/- SD.

Supplemental Experimental Procedures

Mice

All animal procedures were performed in compliance with Cancer Research UK (CRUK) animal ethics committee and UK Home Office guidelines. NOD-SCID IL2Rg^{null} (NSG) and C57BL/6 strains (All Jackson Laboratory, Bar Harbor, Maine, USA), were bred at CRUK's Biological Resources Unit in individually vented cages (IVCs) under Specific Pathogen Free (SPF) conditions. Sub-lethal irradiation was carried out at 3.75Gy with a 137Cs 637 irradiator.

Human HSPCs

Umbilical cord blood was obtained from the Royal London Hospital (London, UK) after informed consent via a protocol approved by the East London Research Ethics Committee and carried out in accordance with the principles of the Helsinki declaration. Mononuclear cells (MNCs) were obtained by centrifugation over a Ficoll-paque gradient (GE Healthcare) and mature cells removed by immunomagnetic depletion using StemSep® Human Progenitor enrichment cocktail (Stem Cell Technologies, Vancouver, Canada). Lineage negative cells were stained with antibodies against Lin, CD34 and CD38 (Clone HB-7) to define HSC- and HPC-enriched populations and further with CD45RA, CD90, CD49f (all BD Biosciences) and the dye Rhodamine123 (Thermo Fisher Scientific~~Life Technologies~~) to obtain highly purified HSCs and sorted by flow cytometric activated cell sorting (FACS).

Mouse HSPCs

C57Bl/6 BM was flushed from femurs and tibias and through crushing of iliac crests and vertebrae in phosphate-buffered saline (PBS) with 2% fetal calf serum (FCS, PAA Labs), heparin and DNAse (Sigma Aldrich). Red blood cells were lysed and nucleated cells were depleted of mature cells using EasySep Mouse Hematopoietic Progenitor kit (Stem Cell

Technologies). Lineage depleted cells were stained with Lin, Sca1, cKit, CD150 and CD48 (BD Biosciences) before FACS sorting.

Labeling, transplantation and imaging of HSC/HPCs for tracking

Following purification, HSPCs were washed twice with PBS before incubating with 2 microM of CFSE for 10 minutes, •••••• microM of•••••• for 20 minutes at 37°C (both dyes from Thermo Fisher ScientificLife Technologies, UK). After incubation, 1ml of ice-cold serum was added before washing and suspending in 2% FCS in PBS. Approximately 2x10^{5} Lin^{-}/CD34^{+}/CD38^{-} (+/-) and 1x10^{6} Lin^{-}/CD34^{+}/CD38^{+} (+/+), 1x10^{5} Lin^{-}/CD34^{+}/CD38^{-}/CD90^{+}/CD45RA^{-}/Rhodamine-Low (+/-/+/-/lo) cells, 5x10^{4} Lin^{-}CD34^{+}/CD38^{-}/CD90^{+}/CD45RA^{-}/CD49f^{+} (CD49f^{+}), 2.5x10^{4} Lin^{-}/Sca1^{+}/cKit^{+}/CD48^{-}/CD150^{+} (LSK SLAM^{+}), 2.5x10^{5} Lin^{-}/Sca1^{+}/cKit^{+}/ not CD48^{-}CD150^{+} (LSK SLAM^{-}), cells per recipient were transplanted IV into 3.75Gy irradiated NSG. Identical numbers of stem-enriched and progenitors were not injected because the homing of progenitors is less efficient than stem-enriched populations. Immediately prior to imaging, streptavidin conjugated Quantum dots (Qdots)-655 (Thermo Fisher ScientificLife Technologies) were IV injected to label endothelial cells. Mice were anesthetized with 2.5% Isoflurane, the head shaved and held in a stereotaxic skull holder. A skin incision revealed the calvaria and methylcellulose (4%, Sigma Aldrich) was applied to prevent the tissue drying. Each mouse was imaged for 4-6 hours before culling, and a separate recipient was used for each time-point. Images were obtained on a Zeiss 710 NLO laser scanning multiphoton microscope with a 20x 1.0 NA water immersion lens. The microscope is equipped with a MaiTai “High Performance” fully automated 1-box mode-locked Ti:Sapphire laser with DeepSee dispersion compensation (Spectra-Physics), tuned to 800nm excitation wavelength. Bone signal- (Second Harmonic Generation signal) was collected at 380-485nm, CFSE at 500-550nm, SNARF at 555-625 and qDots-655 at 640-690nm by non-descanned detectors.

In vivo assays to assess the impact of cell dyes on hematopoiesis

To assess engraftment, 100,000 Lin^- cells stained with CFSE or SNARF and unstained cells were injected intra-bone into 3.75Gy irradiated NSG mice. After 12 weeks, BM was extracted and stained with antibodies recognizing human CD45-PeCy7, mouse CD45-PerCP, human CD3-FITC, human CD19-APC and human CD33-PE (All BD Biosciences) and sorted by flow cytometry. Sorted human cells were IV injected into secondary NSG mice. After a further 12 weeks, BM was analyzed by flow cytometry using the same antibodies previously used to assess primary engraftment.

In vitro colony forming assay and _In vivo_ Limiting Dilution Assay (LDA) to assess functionality of transplanted stem cells

To estimate colony-forming ability, $CFSE^+$ cells were FACS sorted and embedded into methylcellulose with SCF, GM-CSF, IL-3 and EPO (H4434, Stem Cell Technologies). In addition, remaining CFSE labeled +/- sorted cells were injected IV into one irradiated NSG mice. After transplantation, BM was extracted from femurs, tibias and iliac crests and $CFSE^+$ cells re-sorted before adding to methylcellulose. Colonies were counted, washed and re-plated to generate secondary colonies. To estimate stem cell frequency, freshly sorted CFSE labeled +/- cells (“before transplantation”) and cells extracted from the BM of mice transplanted with CFSE labeled +/- cells (“after transplantation”) were injected intra-bone into sublethally irradiated NSG recipients. Engraftment was assessed by FACS 12 weeks after transplantation and was scored positive if BM contained multilineage engraftment above 0.1%. LDA was calculated with ELDA: Extreme Limiting Dilution Assay software (Hu and Smyth, 2009).

Table of all antibodies used in this study

Antibody Type	Clone	Company	Cat number

Human Lin FITC	Mix of different Ab	BDbiosciences	340456
Human CD34 APC	581	BDbiosciences	555824
Human CD38 PE	HB7	BDbiosciences	345806
Human CD38 PeCy7	HB7	BDbiosciences	335825
Human CD45 PeCy7	HI30	eBiosciences	557748
Human CD3 FITC	HIT3a	BDbiosciences	555339
Human CD33 PE	WM53	BDbiosciences	555450
Human CD19 APC	HIB19	BDbiosciences	555415
Human CD49f	GoH3	BDbiosciences	555736
Human CD45RA FITC	HI100	BDbiosciences	555489
Human CD90 APC	5E10	eBiosciences	17-0900-42
Mouse CD45 PerCP	30-F11	BDbiosciences	557235
Mouse CD45 PeCy5	30-F11	BDbiosciences	553082
Mouse CD150	TC15-12F12.2	Biolegend	115912
Mouse CD48 FITC	HM48-1	BDbiosciences	557484
Mouse cKIT APC	2B8	eBiosciences	1171-83
Mouse SCA1 PeCy7	D7	eBiosciences	25-5982-82
Mouse Lin	Mix of different Ab	StemCell Technologies	19756
StreptAvidin PerCP	-	BDbiosciences	554064

Additional References

Hu, Y., and Smyth, G.K. (2009). ELDA: extreme limiting dilution analysis for comparing depleted and enriched populations in stem cell and other assays. Journal of immunological methods *347*, 70-78.

Video Legends

Video 1. A single z-section showing Human +/- (green) and +/+ (red) cell migration at day 3 after transplantation. Endothelial cells on blood vessels are shown in magenta. Scale bar = 40μm.

Video 2. A single z-section showing Human +/- (green) and +/+ (red) cell migration at day 4 after transplantation. Endothelial cells on blood vessels are shown in magenta, and the bone surface in cyan. Scale bar = 50μm.

Video 3. A single z-section showing a Mouse SLAM^{+} cell (green) migrating at 16 hours after transplantation. Endothelial cells on blood vessels are shown in magenta and the bone surface in cyan. Scale bar = 50μm.

Video 4. A single z-section showing a Mouse LSK^{+} cell (red) migrating at 16 hours after transplantation. Endothelial cells on blood vessels are shown in magenta and the bone surface in cyan. Scale bar = 40μm.

Video 5. A single z-section showing a human +/- cells in green 4 days after transplantation and IV injection of Bio5192. The bone surface is shown in cyan and auto-fluorescence in orange. Scale bar = 40μm.

Video 6. A single z-section showing human +/- cells 4 days after transplantation and IV injection of AMD3100. Endothelial cells are shown in magenta. Scale bar = 40μm.
