# Supplementary figures and images for: Different Motile Behaviors of Human Hematopoietic Stem versus Progenitor Cells at the Osteoblastic Niche
Source: Stem Cell Reports. 2015 Oct 8;5(5):690–701. doi: 10.1016/j.stemcr.2015.09.003 (PMC4649139; doi:10.1016/j.stemcr.2015.09.003)

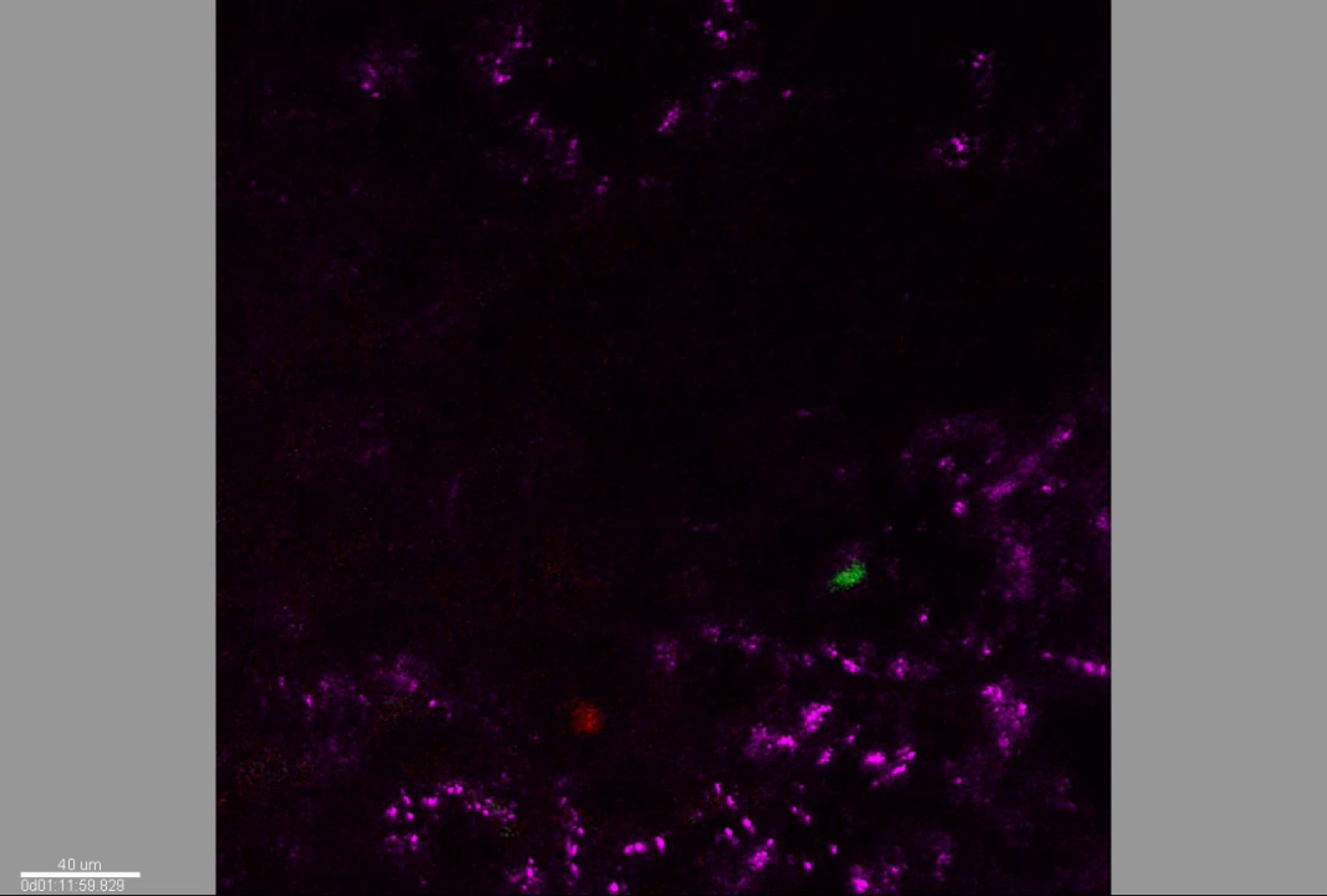

Supplement: Movie S1. A Single Z Section Showing Human +/−, which Is Green, and +/+, which Is Red, Cell Migration at Day 3 after Transplantation — Endothelial cells on blood vessels are shown in magenta. Scale bar represents 40 μm. [file mmc2.jpg]

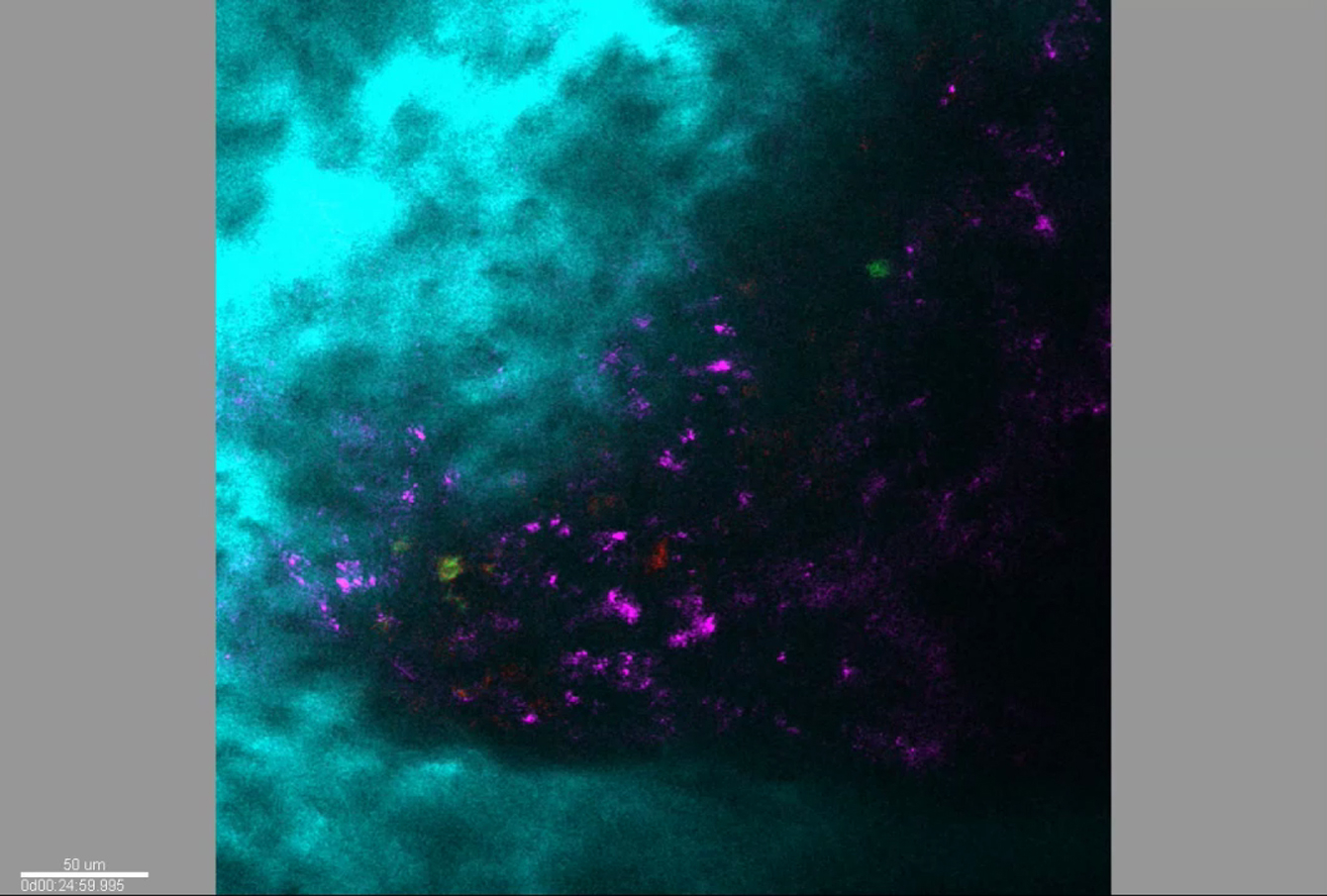

Supplement: Movie S2. A Single Z Section Showing Human +/−, which Is Green, and +/+, which Is Red, Cell Migration at Day 4 after Transplantation — Endothelial Cells on Blood Vessels Are Shown in Magenta, and the Bone Surface in Cyan. Scale bar represents 50 μm. [file mmc3.jpg]

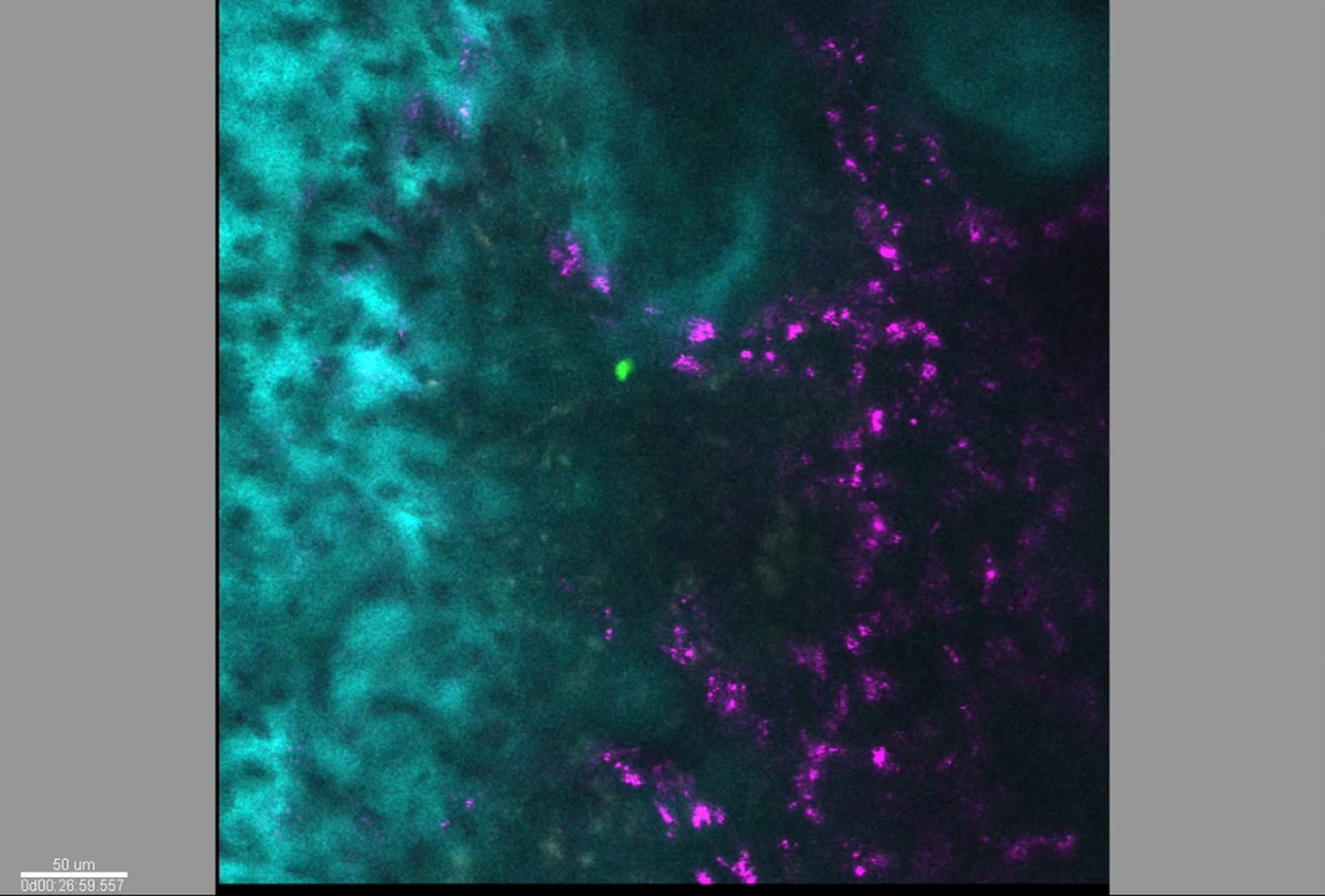

Supplement: Movie S3. A Single Z Section Showing a Mouse SLAM+ Cell, in Green, Migrating at 16 hr after Transplantation — Endothelial cells on blood vessels are shown in magenta, and the bone surface is in cyan. Scale bar represents 50 μm. [file mmc4.jpg]

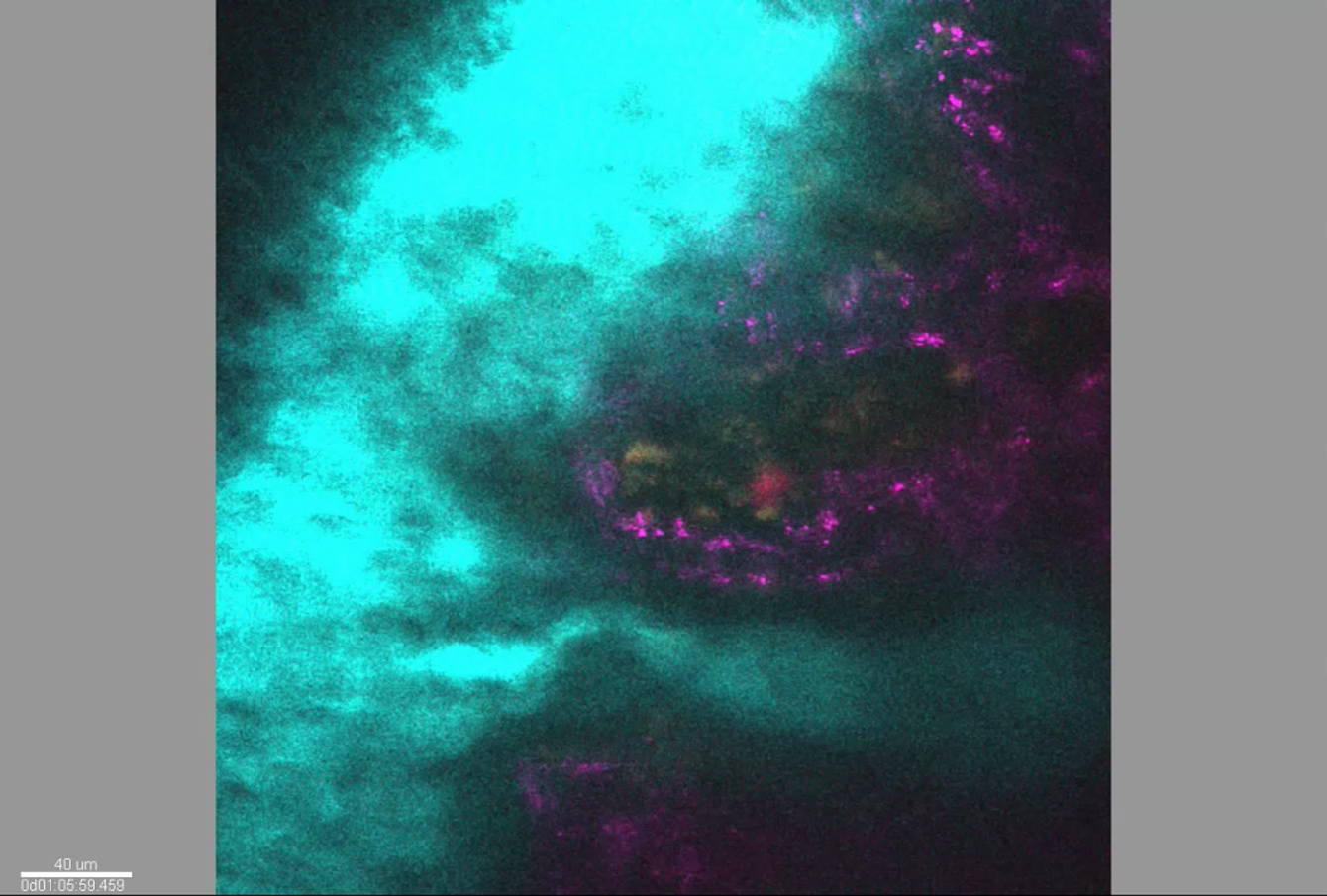

Supplement: Movie S4. A Single Z Section Showing a Mouse LSK+ Cell, in Red, Migrating at 16 hr after Transplantation — Endothelial cells on blood vessels are shown in magenta, and the bone surface is in cyan. Scale bar represents 40 μm. [file mmc5.jpg]

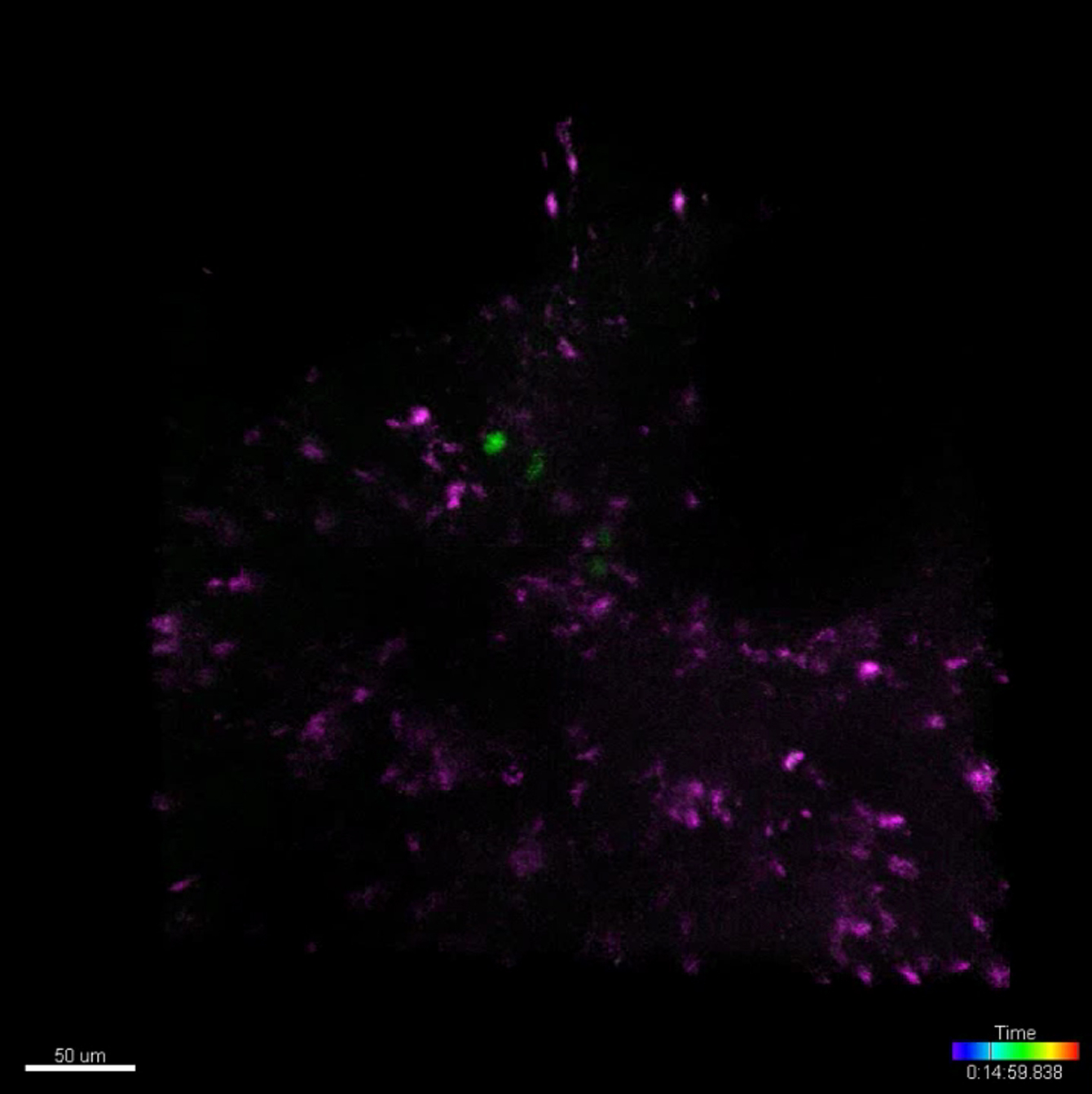

Supplement: Movie S5. A Single Z Section Showing Human +/− Cells in Green 4 Days after Transplantation and IV Injection of Bio5192 — The bone surface is shown in cyan and autofluorescence in orange. Scale bar represents 40 μm. [file mmc6.jpg]

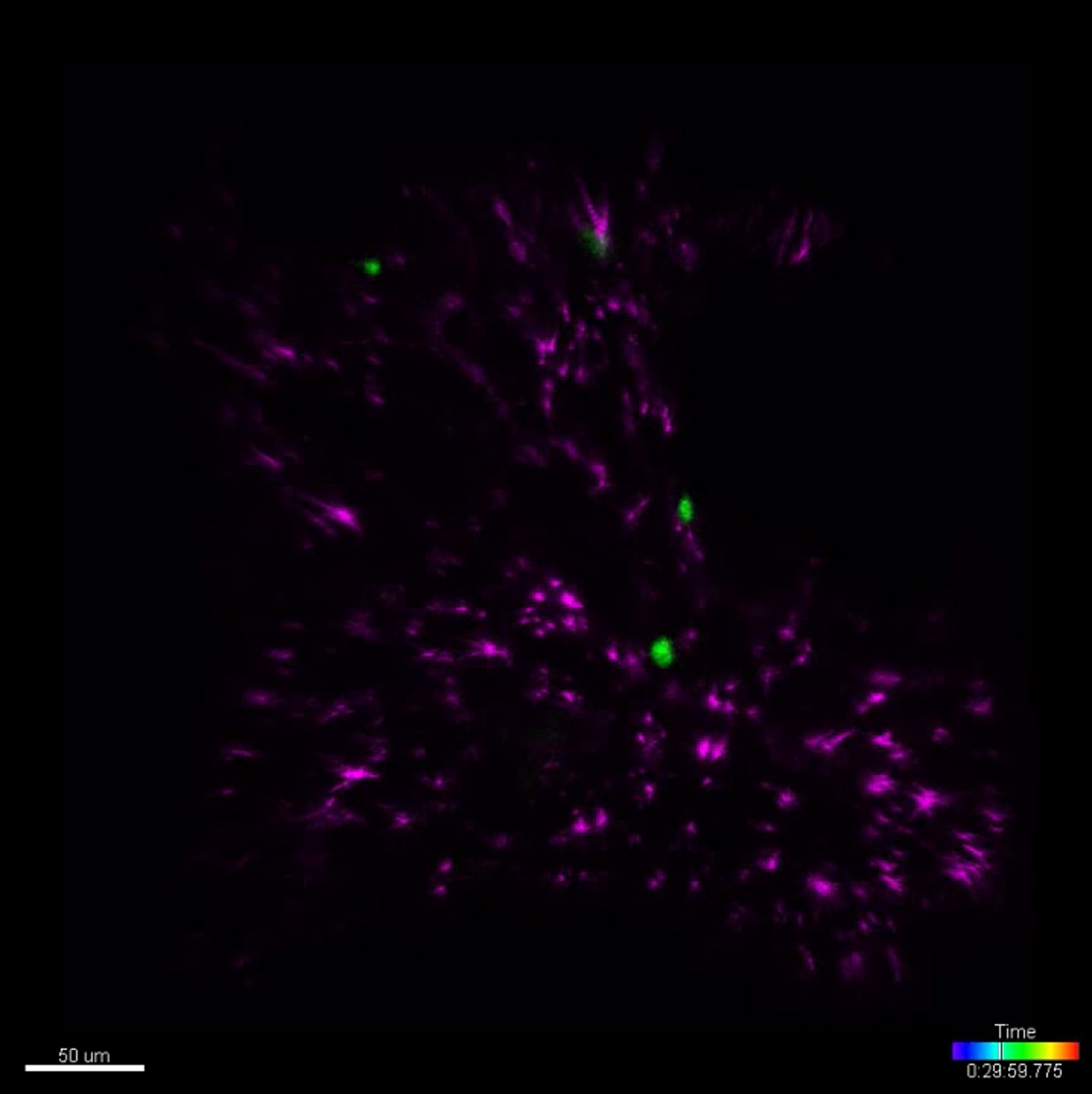

Supplement: Movie S6. A Single Z Section Showing Human +/− Cells 4 Days after Transplantation and IV Injection of AMD3100 — Endothelial cells are shown in magenta. Scale bar represents 40 μm. [file mmc7.jpg]
