# Supplementary material for: Different Motile Behaviors of Human Hematopoietic Stem versus Progenitor Cells at the Osteoblastic Niche
Source: Stem Cell Reports. 2015 Oct 8;5(5):690–701. doi: 10.1016/j.stemcr.2015.09.003 (PMC4649139; doi:10.1016/j.stemcr.2015.09.003)
Supplement: Document S2. Article plus Supplemental Information [file mmc8.pdf]

Stem Cell Reports
Article
OPEN ACCESS

Different Motile Behaviors of Human Hematopoietic Stem versus Progenitor Cells at the Osteoblastic Niche

Katie Foster,[1] François Lassailly,[1] Fernando Anjos-Afonso,[1] Erin Currie,[1] Kevin Rouault-Pierre,[1] and Dominique Bonnet[1,*]
[1]Haematopoietic Stem Cell Laboratory, The Francis Crick Institute, London WC2A 3LY, UK
*Correspondence: dominique.bonnet@crick.ac.uk
http://dx.doi.org/10.1016/j.stemcr.2015.09.003
This is an open access article under the CC BY license (http://creativecommons.org/licenses/by/4.0/).

SUMMARY

Despite advances in our understanding of interactions between mouse hematopoietic stem cells (HSCs) and their niche, little is known about communication between human HSCs and the microenvironment. Using a xenotransplantation model and intravital imaging, we demonstrate that human HSCs display distinct motile behaviors to their hematopoietic progenitor cell (HPC) counterparts, and the same pattern can be found between mouse HSCs and HPCs. HSCs become significantly less motile after transplantation, while progenitor cells remain motile. We show that human HSCs take longer to find their niche than previously expected and suggest that the niche be defined as the position where HSCs stop moving. Intravital imaging is the only technique to determine where in the bone marrow stem cells stop moving, and future analyses should focus on the environment surrounding the HSC at this point.

INTRODUCTION

Coordinating the balance between hematopoietic stem cell (HSC) quiescence and self-renewal is crucial for maintaining lifelong hematopoiesis and is controlled by a complex network of intrinsic and extrinsic signaling interactions with the microenvironment. While our understanding of the regulators controlling mouse hematopoietic stem/progenitor cells (HSPCs) has increased (reviewed in Morrison and Scadden, 2014), little is known about whether these factors and cellular micro-environmental component(s) that are important for mouse HSPCs could also be extrapolated to human HSPCs. The most widely used system that mimics the human niche in vivo is the xenotransplantation model. In this system, immunodeficient mouse bone marrow (BM) provides efficient support of human HSPCs allowing multilineage reconstitution. Once transplanted, HSPCs are home to the BM where they reside in specific niches that direct proliferation, quiescence, apoptosis, and mobilization into the periphery. Reconstitution can be followed by peripheral blood sampling or BM aspiration weeks after transplantation, but the first and most critical stages of lodgment (defined as their position at early time points post-transplant; Lapidot et al., 2005) are not well characterized. A recent study provided the first demonstration of the use of human-mouse xenografts as a surrogate model to study positioning of human HSPCs in human bone biopsy specimens, indicating that similar micro-environmental niches could be defined in the xenotransplant model (Guezguez et al., 2013). However, current approaches visualizing stem cells and their niche in fixed sections cannot define the true niche since the cell may still have been migrating when the tissue sample was taken. The only way to visualize cell movements in the BM with sufficient spatial/temporal resolution without physically damaging the niche is by intravital imaging of the calvaria (Lo Celso et al., 2009). While different in structure and developmental origin to the long bones, HSCs in the calvaria show identical HSC frequency and function to those found in the femur (Lassailly et al., 2013; Lo Celso et al., 2009).

Intravital imaging of mouse HSPCs in calvaria showed that by 16 hr after transplantation, the majority of cells had entered the bone, crossed the endothelium, and lodged within a few cell diameters of bone. HSPCs localized to distinct regions according to their differentiation status (Lo Celso et al., 2009); at least in the calvaria, both osteoblastic and vascular niches are not physically separate, and a cell can be located within both. However, it remains unclear whether we can extrapolate the definition of the mouse HSC niche to human. In order to study the early phases of human HSPC homing and lodgment, we adopted a similar approach used by Lo Celso et al. (2009) to track human and mouse HSPCs in the calvaria of live mice. Using time-lapse imaging, we show that both human and mouse HSCs and hematopoietic progenitor cells (HPCs) exhibit strikingly different motile behaviors. It takes human HSC-enriched cells longer than expected to find their niche at which point motility decreases. In comparison, progenitor-enriched populations continue to navigate the micro-environment. We show that blocking integrin binding within the niche can reverse the relatively non-motile phenotype of HSCs, indicating a role for integrins in the motility of HSCs after lodgment. We also found a similar

pattern of motility in mouse HSPCs, where HSCs were much less motile than HPCs. Our results suggest that for human cells, the definition of lodgment should be described by their position when cells stop moving and that, on a whole-population level, migratory behavior in vivo can be used to identify a more pure population of human and mouse HSCs. Imaging of migratory behavior is crucial in order to pinpoint the location of where stem cells stop moving and lodge, which is indicative of the HSC niche. Once the location is confirmed, a more thorough analysis of the microenvironment at this location can be performed. Moreover, intravital imaging can be used to test pharmacological, antibody blocking, or genetic manipulation of molecules thought to play a role in HSC function since it allows one to determine whether functional defects occur from an inability to migrate or adhere during the distinct homing, lodgment, and mobilization processes.

RESULTS

Developing Tools to Track Human/Mouse HSPCs by Intravital Microscopy

In order to visualize HSPC interactions with their niche, cells must be labeled with a fluorescent marker. Lipophilic dyes have been used to track HSPCs, but leakage of dye and contamination of microenvironment is possible (Lassailly et al., 2010). Amine reactive cell dyes do not appear to leak and are frequently used to track lymphocyte division and migration (De Clerck et al., 1994; Nolte et al., 2004). However, since CFSE and SNARF have been reported to have some negative effects on cell function, we performed analyses to find the optimal concentration (carboxyfluorescein succinimidyl ester [CFSE] 2 μM and SNARF 1.5 μM, data not shown). We confirmed non-toxicity using in vivo transplantation experiments, showing no detrimental effects on engraftment of dye-labeled HSPCs in primary and secondary recipients (Figures S1A and S1B). Furthermore, each time point and population were repeated using both dyes in order to ensure no effect on motility of a specific dye (Figure S1C). The possibility of autofluorescent cells in the BM being mistaken for dye-labeled cells is minimal since autofluorescent cells appear in both CFSE (green) and SNARF (red) detectors and appear as yellow/orange objects (Figure S1D). Any object that appears in both CFSE and SNARF channels was excluded from the analysis.

In addition to visualizing stem and progenitor populations labeled with dyes, we collected the second harmonic signal emitted by collagen in bone (cyan). The distance from the center of the cell to the closest pixel of bone surface is displayed graphically. Using this protocol, we are able to track cell position relative to the bone surface and movements over time.

Human HSC- and HPC-Enriched Populations Take up to 4 days to Lodge in an Endosteal Niche

A recent report from Bhatia's group showed that human HSPCs xenotransplanted into an immunodeficient mouse resided in both vascular and endosteal regions at steady state, similar to indigenous HSPCs in human bone. The report by Bhatia's group demonstrated functional human hematopoiesis in mice, validating the use of human-mouse BM xenografts. However, the authors did not track cell migration over time in order to describe homing and lodgment (Guezguez et al., 2013). We applied a similar xenotransplantation technique to study migration, homing, and lodgment of human HPSCs. Prior to starting these experiments, we tested our intravital imaging technique using mouse HSPCs to confirm expected results with those previously published (Lo Celso et al., 2009). Likewise, we saw that at 16 hr after transplantation mouse HSPCs had crossed the endothelium to enter the BM and were located with two to three cell diameters of the bone surface (data not shown). To track human HSPCs, we used expression of lineage markers (Lin), CD34 and CD38 to sort HSC-enriched $Lin^{-}CD34^{+}CD38^{-}$ and HPC-enriched $Lin^{-}CD34^{+}CD38^{+}$ cells (herein referred to as +/− and +/+). We transplanted these cells into sublethally irradiated NSG mice. By 16-hr post-transplant, the cells have crossed the endothelium and entered the BM cavity (Figure 1B, top row). However, time-lapse microscopy indicated that cells were still motile, suggesting that at 16 hr they may not be lodged in their final niche, so we continued to track cells for up to 4 days after transplantation (Figures 1A and 1B). Serial analyses showed that both +/− and +/+ populations continue to move and get progressively closer to bone (Figures 1A and 1B), eventually settling at an average distance of within one to two cell diameters (one cell diameter = 6 to 8 μm; Xie et al., 2009) of the endosteum at day 4 (Figures 1A and 1B, bottom row). It is possible that differences in position of cells over time are due to changes in niche architecture such as vascular remodeling or osteoblast loss following irradiation. As such, a more detailed analysis of transplanted cell position in relationship to other niche components after irradiation will be required. Another possible explanation for the change in position of HSPCs is that cells that are close to the endosteum persist, while those located further away are lost through apoptosis and/or have proliferated and thus lost their dyes. However, it is not currently possible to measure apoptosis of cells that reside in one location versus another in order to address this question. Furthermore, we could not detect cells that have undergone proliferation.

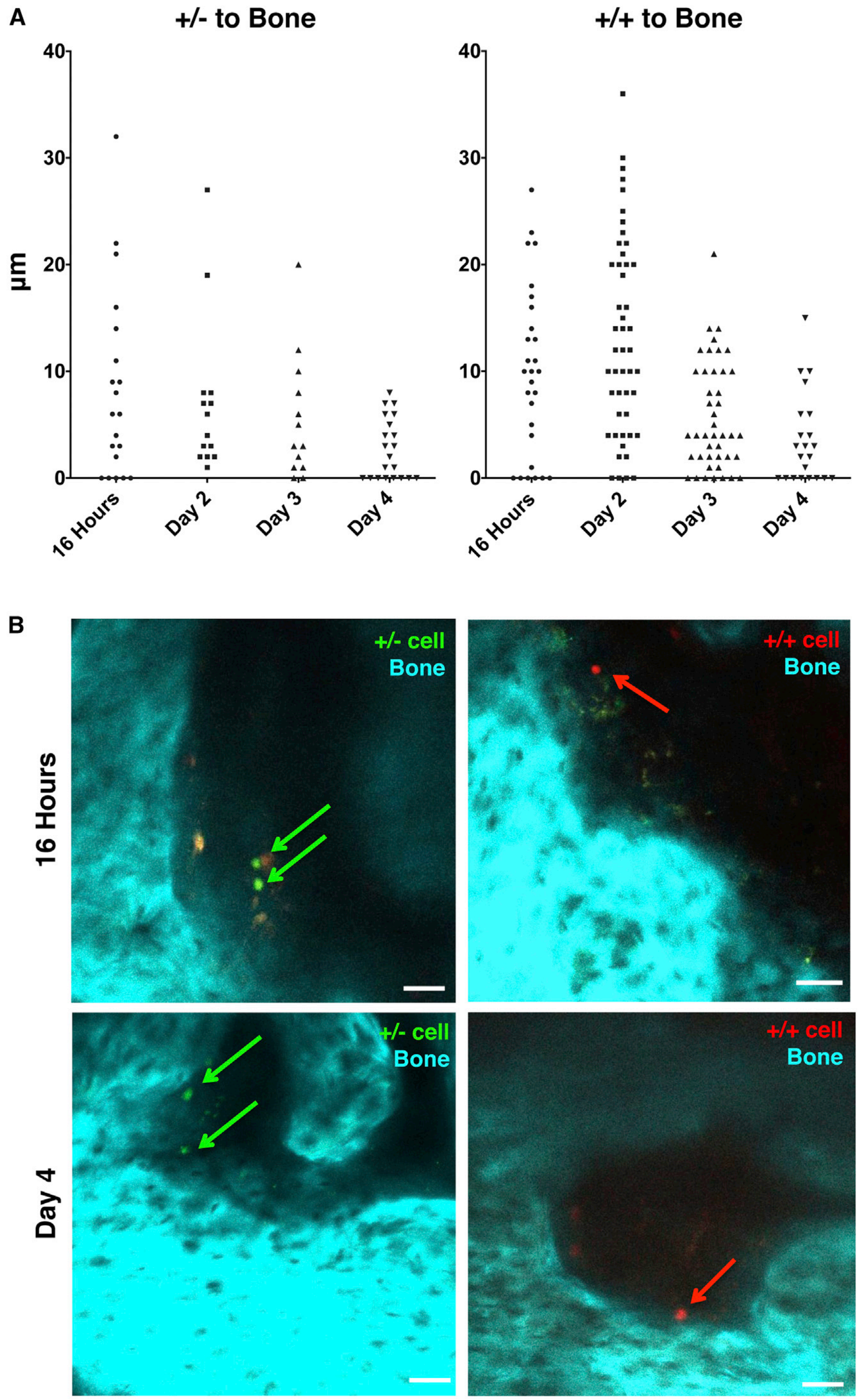

Figure 1. In Vivo Imaging of Human HSPCs

CB mononuclear cells (CB MNCs) were flow sorted to purify Lin−$CD34^+CD38^-$ (+/−) and $Lin^-CD34^+CD38^+$ (+/+) populations and then labeled with dyes and injected IV in NSG recipients.

(A) Live distance measurements in µm of +/− and +/+ cells in the calvaria to bone at 16 hr and days 2, 3, and 4 post-transplantation.

(B) Live images from calvaria of mice transplanted with +/− (green, left) and +/+ (red, right) cells at 16 hr (top) and day 4 (bottom) after transplantation. Second harmonic signal generated by the bone is cyan. Scale bars represent 50 µm. N = 9 recipients of cells from pooled CB units (two recipients for 16 hr, two recipients for day 2, three recipients of day 3, three recipients of day 4). Each recipient received cells from approximately six to nine CB donors.

HSCs that Home to the BM and Persist after Transplantation Retain Stem Cell Activity

It is possible that at 4 days after transplantation the cells that we are imaging may no longer be stem cells and have differentiated and/or lost repopulating ability. Therefore, the phenotype of transplanted cells we see may not be characteristic of stem cells. In order to determine the extent of transplanted cell differentiation, we retrieved the $CFSE^+$ cells at day 4 and performed a FACS analysis of these cells. A high proportion of these $CFSE^+$ cells were still $CD34^+CD38^-$, and more than 66% were $CD45RA^-CD90^+$, indicating that these $CFSE^+$ cells imaged at day 4 are still phenotypically enriched in repopulating cells (Figure 2A). To confirm this functionally, we compared their secondary

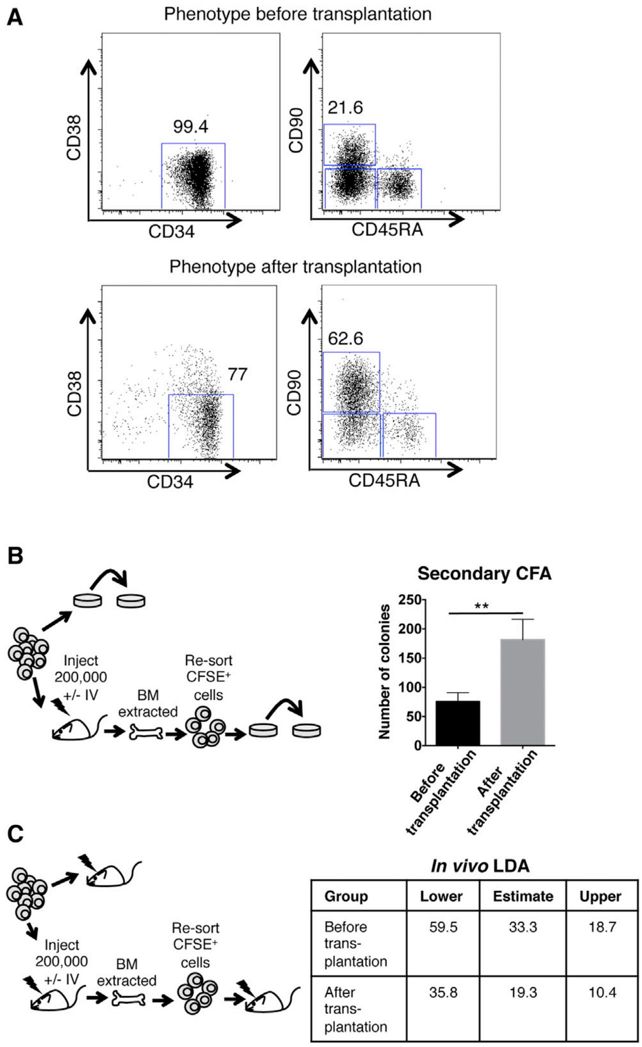

Figure 2. Estimation of Stem Cell Frequency of Cells that Home to and Persist in the BM after Transplantation

(A) FACS analysis (top) of the CSFE$^+$ CD34$^+$CD38$^-$ before transplantation (bottom) FACS analysis of the CSFE$^+$ cells extracted from the BM of NSG mice at day 4 post-transplantation.

(B) (left) Protocol to assess colony-forming ability of cells visible by microscopy; 300 Lin$^-$CD34$^+$CD38$^-$ (+/−) cells were plated directly into methylcellulose (before transplantation group) or stained with CFSE and injected IV into NSG recipient. After homing, mice were sacrificed; BM was extracted from femurs, tibias, and iliac crests and re-sorted to obtain CFSE$^+$ cells; and these were plated into methylcellulose (after transplantation group). 75,000 cells from primary colonies were obtained after 14 days and re-plated to generate secondary colonies. (Right) Number of secondary colonies. N = 5 independent experiments from pooled CB units. Error bars represent ± SD. *$p < 0.05$, **$p < 0.01$, ***$p < 0.001$.

(C) (left) Protocol to assess stem cell frequency after transplantation. Lineage-depleted CB MNCs were stained with Lin, CD34, and CD38 antibodies and sorted by FACS to purify Lin$^-$CD34$^+$CD38$^-$ cells. Cells were injected intra-bone at limiting dose into sublethally irradiated recipients (before transplantation group) or stained with CFSE and injected IV into NSG recipient. After homing, mice were sacrificed, BM was extracted from femurs, tibias, and iliac crests and re-sorted by FACS to obtain CFSE$^+$ cells and were injected intra-bone into the tibia at limiting dilution in sublethally irradiated recipients (after transplantation group). (Right) Engraftment at 12 weeks was assessed by flow cytometry for human CD45 multilineage (containing CD3, CD19, and CD33 cells) engraftment and scored positive if above 0.1%. ELDA software was used to calculate the frequency of repopulating cells. N = 48 mice (before and after cohort of mice used per condition: two mice each for 5, 20, and 40 cell doses, six mice each for 60 and 80 cell dose, from two independent experiments from approximately 9–12 pooled CB units).

clonogenic capacity by colony forming unit (CFU) of the CFSE$^+$ cells that had been transplanted and recovered by fluorescence-activated cell sorting (FACS) ("after transplantation") to fresh CFSE labeled +/− cells ("before transplantation") that were plated immediately into methylcellulose. After 2 weeks, the colonies were re-plated to generate secondary colonies (Figure 2B). The group of cells that was extracted from the mouse after transplantation showed a 2- to 3-fold increase in secondary re-plating ability (75,000 cells from "before transplantation" colonies gave 75 colonies' 75,000 cells from "after transplantation" colonies gave 181 colonies; Figure 2B). We further performed an in vivo limiting dilution assay (LDA) experiment to assess stem cell frequency in CFSE$^+$ cells before and after transplantation. Before transplantation and after transplantation cells were injected intra-bone into the tibia of sublethally irradiated NSG mice at doses ranging from 5 to 80 cells (Figure 2C, left). After 12 weeks, engraftment was assessed by flow cytometry and repopulating-cell frequency calculated. The engraftment frequency after transplantation (frequency: 1 in 19, 95% confidence interval: 1 in 10.4 to 1 in 35.8) was statistical similar to that before transplantation (frequency: 1 in 33.3, 95% confidence interval: 1 in 18.7 to 1 in 59.5) (Figure 2C, right). These analyses showed that CFSE$^+$ cells imaged at day 4 were still functionally enriched in cells with repopulating and multipotential activity, suggesting that we are still imaging bona fide stem-enriched cells.

HSC- and HPC-Enriched Populations Exhibit Distinct Motility Behaviors

Since HSCs appeared to keep moving after transplantation, we performed live cell tracking to determine whether and/or when the cells stop migrating and whether there are any dynamic differences during their homing. At 16 hr, days 2, 3, 4, and 6 after transplantation, we tracked cells for 2 to 4 hr and calculated the displacement per hour, speed, and straightness. Displacement per hour describes the distance between the start and end point of migration corrected for time, which shows overall movement of the cell.

Straightness is a measure of whether a cell is moving in a straight line (directed motion) or moving randomly; a value closer to 1 equals a straight path, while a lower value is indicative of random walk. Displacement values showed that 16 hr after transplantation, there were no differences between +/− and +/+ populations (Figure 3Ai). Both populations have an elongated morphology and are highly motile by day 3 (Figure 3Ai; Movie S1). However, by day 4 after transplantation, +/− cells dramatically decrease their displacement and remain rounded while the majority of +/+ cells remain highly motile and elongated (Figure 3Ai; Movie S2), and although there is a smaller subpopulation that are still moving, the majority of +/− cells stay within a few microns of their starting position. Statistical analysis by ANOVA test showed a significant difference in displacement between +/+ and +/− cells ($p = 0.001$) and between time points ($p = 10^{-06}$) (comparing days 3 and 4). The difference in motility is highlighted when migration tracks are displayed graphically; the tracks of +/− (green) and +/+ (red) cells show similar patterns at day 3 (Figures 3D, left, and S2A and S2B), while the same analyses show a marked difference in pattern at day 4 (Figures 3D, right, and S2A and S2B). Analysis of cell speed showed a similar pattern whereby both had a similar speed at day 3, but at day 4, +/− cells had a lower speed than +/+ cells. Statistical analysis by ANOVA test showed a significant difference in speed between +/+ and +/− cells ($p = 10^{-05}$) and between time points ($p = 10^{-09}$) (comparing days 3 and 4) (Figure 3Bi). The pattern of migration was also changed significantly over days 3 and 4, with +/− cells exhibiting a more random walk type of motility by day 4, as indicated by lower straightness values (Figure 3Ci) ($p = 10^{-05}$).

Observations of cell movements indicated that +/− cells changed their morphology between days 3 and 4 after transplantation. In order to determine whether there was a significant difference in cell shape, we measured the sphericity of HSPCs (+/− cells) when they were at their most motile on Day 3 and at Day 4, when the majority of cells stopped moving. These analyses showed that +/− cells at day 3 are statistically less spherical (a value closer to 0) and thus more polarized compared with +/− cells at day 4, which were more spherical (a value closer to 1; Figure 3E). Together, the change in morphology combined with a difference in motility, as measured by displacement, speed, and straightness, indicates a change in behavior of the majority of the +/− population over time.

To determine whether the distinct pattern of +/− motility persists beyond 4 days, we imaged 2 days later at day 6. Hardly any +/+ cells were observed at this later time point, probably due to high proliferation of the cells and dilution of the dye, or apoptosis, and are not included in the data. On a whole population level, the +/− population was more motile at day 6 than day 4 (Figure 3Ai). However, on closer inspection, it appears that there are two distinct subpopulations; one that remains non-motile and now a larger population of motile cells. Indeed, further analysis of speed and straightness parameters showed that there was no difference in speed between days 4 and 6 (Figure 3Bi), but an increase in straightness, whereby cells were exhibiting a more directed motion type of movement (Figure 3Ci). In order to determine whether differences between HSC and HPC motility were specific to human HSPCs, we also tracked the migration of mouse HSPCs. We FACS-sorted mouse $Lin^{-}SCA1^{+}cKIT^{+}CD48^{-}CD150^{+}$ (LSK $SLAM^{+}$, HSC) and the rest of the $Lin^{-}SCA1^{+}cKIT^{+}$ (LSK) cells that were not $CD150^{+}CD48^{-}$ (this include HPC1, HPC2, and MPPs following Sean Morrison's group definition; Oguro et al., 2013). For simplicity, herein we call these cells (LSK $SLAM^{-}$, HPC). Because of high HPC proliferation, we could only track LSK $SLAM^{-}$ cells for 16 hr. Similar to human HSPCs, mouse HSCs exhibited a reduced displacement and had a rounded morphology (Figure 3Aii; Movie S3) compared with their HPC counterparts, which were highly motile and elongated (Figure 3Aii; Movie S4). Analysis of speed indicated a significant difference between LSK $SLAM^{+}$ and LSK $SLAM^{-}$ cells (Figure 3Bii; Movies S3 and S4), and LSK $SLAM^{-}$ cells exhibited a trend, but not significant difference, toward more directed motion pattern of motility as measured by straightness (Figure 3Cii; Movies S3 and S4), indicating that the differences between stem and progenitor cell motility is consistent between species and not specific to human cells in the xenotransplantation setting.

Heterogeneity in Motile Behavior Is Not Accounted for by Stem Cell Purity

As indicated in Figure 3Ai, some heterogeneity exists in the behavior of the +/− stem cell-enriched population where not all the +/− cells stop migrating at day 4. We hypothesized that more highly purified stem cells would be the least displacing, while those still highly migratory were of a more differentiated population. To test this, we sorted highly purified HSC populations based on expression of Lin^{-}, $CD34^{+}$, $CD38^{-}$, $CD90^{+}$, $CD45RA^{-}$, $CD49f^{+}$ (Notta et al., 2011) (herein called $CD49f^{+}$) and $CD34^{+}$, $CD38^{-}$, $CD90^{+}$, $CD45RA^{-}$, and efflux of Rhodamine 123 (Kim et al., 1998) (herein called +/− /+/−/lo). We labeled and transplanted these cells and imaged 4 days later. There were no differences in location between +/−, $CD49f^{+}$, and +/−/+/−/lo populations (Figure S2C). Surprisingly, however, the displacement, speed, and straightness were also similar to +/− cells (Figures 3Aiii, 3Biii, and 3Ciii), and there was still heterogeneity in the motility of cells. This could be due to further heterogeneity in the $CD49f^{+}$ and +/−/+/−/lo phenotypes and thus functional differences that result in a difference in migration. Alternatively,

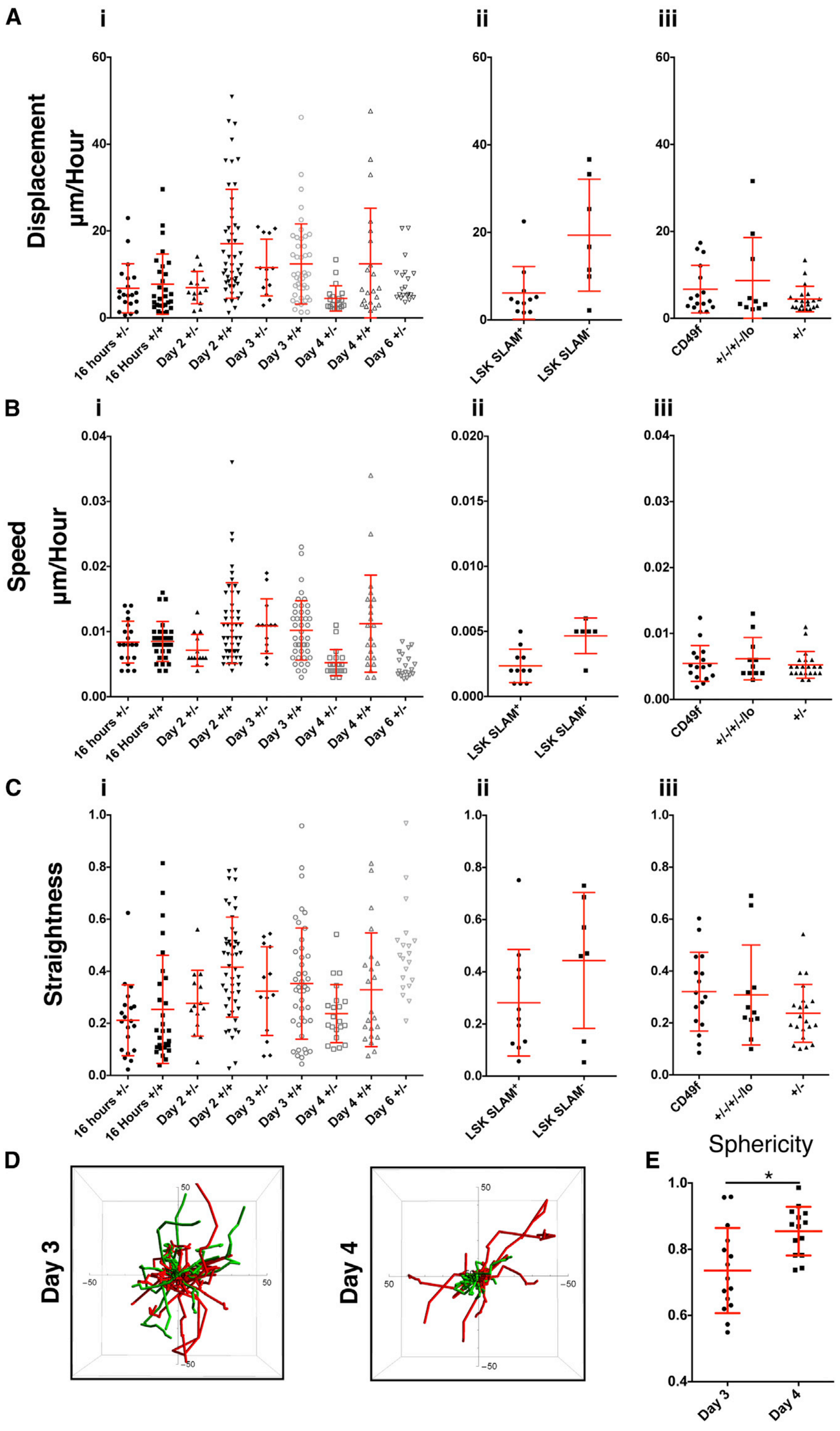

Figure 3. In Vivo Migration of Human and Mouse HSPCs

CB MNCs were lineage depleted, stained with antibodies, and flow sorted to purify $Lin^-CD34^+CD38^-$ (+/−) and $Lin^-CD34^+CD38^+$ (+/+) populations. Cells were labeled and injected IV into NSG recipients.

(A–C) The displacement per hour (A), speed (B), and straightness (C) of (i) +/− and +/+ cells in μm in the calvaria at 16 hr and at days 2, 3, 4, and 6 post-transplantation. N = 10 recipients of cells from pooled CB units (two recipients for 16 hr, two recipients for day 2, three recipients of day 3, three recipients of day 4, and one recipient of day 6). Each recipient received cells from approximately six to nine CB donors. (ii) BM obtained from femurs and tibias of C57Bl/6 mice were lineage depleted, stained with Lin, SCA1, cKIT, CD150, and CD48 antibodies, and flow sorted to obtain LSK $SLAM^+$ (Lin^-, $SCA1^+$, $cKIT^+$, $CD48^-$, $CD150^+$) and LSK $SLAM^-$ (Lin^-, $SCA1^+$, $cKIT^+$, not $CD48^-$, CD150+). The cells were labeled and injected IV into NSG recipients. The displacement per hour (A), speed (B), and straightness (C) of LSK $SLAM^+$ and LSK $SLAM^-$ in the calvaria at 16 hr is shown. N = 2 recipients at each time point of cells from pooled BM from approximately 10 C57Bl/6 donor mice for each recipient. (iii) CB MNCs were lineage depleted; stained with CD38, CD34, CD45RA, CD90, and CD49f antibodies ("CD49f") or the cell dye Rhodamine123 (+/−/+/−/lo); and flow sorted to purify HSCs compared with HSPCs ($CD34^+/CD38^-$). Cells were labeled and injected IV into NSG recipients. Displacement per hour (A), speed (B), and straightness (C) of cells in the calvaria at 4 days after transplantation is shown. N = 3 recipients of $CD49f^+$ cells, N = 2 recipients of +/−/+/−/lo cells, and N = 3 recipients of +/− cells of cells from approximately six to nine pooled CB units for each recipient. The +/− data in this figure are the same data from Figure 3A.

(D) The actual migration tracks for all +/− (green) and +/+ cells (red) from Figure 3A were overlaid on a 3D track plot (Wolfram Mathmatica) for day 3 (left) and day 4 (right). Total track length is displayed which ranges from 1–2 hr per cell.

(E) Sphericity for +/− cells at day 3 and day 4. Data obtained from the same cells in Figure 3A.

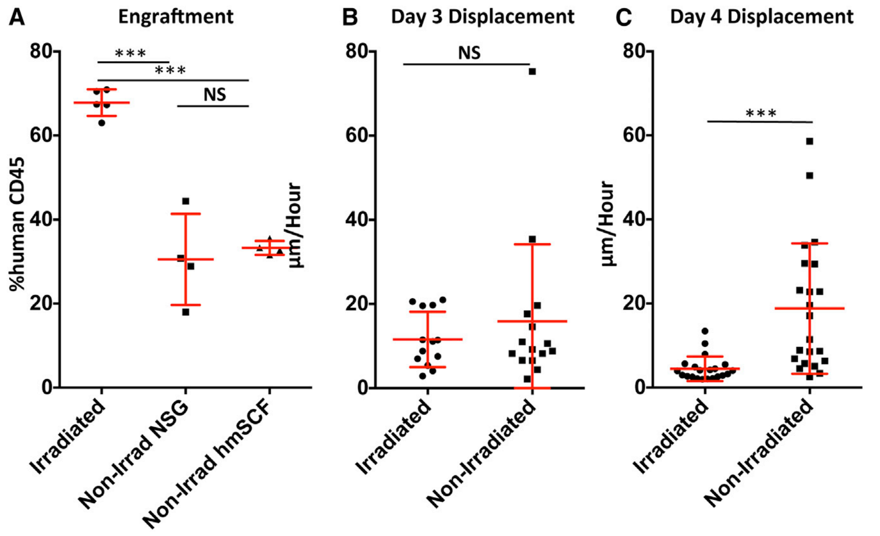

Figure 4. Role of Conditioning Regimes in the Motility Behavior of HSPCs

(A) 100,000 lineage-depleted CB MNCs were IV injected into sublethally irradiated NSG (3.75 Gy 24 hr prior to transplantation), non-irradiated NSG, and non-irradiated hmSCF NSG mice. At 12 weeks after transplantation, BM from tibia, femur, and iliac crests was obtained and stained with human CD45 antibody and analyzed by flow cytometry. Error bars represent ± SD. ***$p < 0.001$. N = 5 irradiated NSG, N = 4 non-irradiated NSG, and N = 4 non-irradiated hmSCF-NSG recipients of pooled CB units.

(B and C) CFSE-labeled FACS-sorted Lin$^-$CD34$^+$CD38$^-$ cells were transplanted IV into 3.75 Gy sub-lethally irradiated NSG (3.75 Gy 24 hr prior to transplantation), non-irradiated NSG, or hmSCF NSG mice. Data indicate displacement per hour of +/− cells in µm in calvaria at 3 (B) and 4 (C) days after transplantation. Error bars represent ± SD. ***$p < 0.001$. N = 3 irradiated mice, N = 4 non-irradiated mice.

it is possible the CD49f$^+$ and +/−/+/−/lo populations are fairly pure and, depending on the cues that the cell receives from the environment following transplantation, have different dynamic properties. In order to further clarify why there is heterogeneity in terms of behavior within even the highly enriched HSC population, it would be necessary to assess the cell-cycle status or gene expression profile of motile versus non-motile cells; however, it is not currently possible to purify the two populations from the BM based on motility. Moreover, even though the dyes used to track cell divisions are routinely used to track cell divisions by flow cytometry, due to differential scattering of reflected light dependent on how deep in the BM the dye-labeled cell is positioned, unfortunately, we could not use the dye to count cell divisions by imaging and thus correlate position with proliferation status.

Could Conditioning Regimes Account for the Differences in HSPC Motility Observed?

Since sublethal irradiation was used to condition the mice for transplantation, it is possible that the difference in motility of HSPCs could be due to changes in the microenvironment and/or in the hematopoietic cells after conditioning. To investigate this possibility, we performed identical experiments into non-irradiated NSG and non-irradiated NSG human membrane bound stem cell factor (hmSCF) mice. NSG-hmSCF mice were recently reported as an improvement to the NSG model, as these mice do not require irradiation to permit engraftment of human hematopoietic cells (Brehm et al., 2012; Takagi et al., 2012). In our hands, we found a significant reduction in engraftment in non-irradiated NSG and NSG-hmSCF recipients compared with irradiated mice; however, there were no differences in engraftment between non-irradiated NSG and non-irradiated NSG-hmSCF recipients (Figure 4A); therefore, the two non-irradiated groups were merged in further analyses. We performed motility comparisons at 3 versus 4 days post-transplantation. At 3 days after transplantation, there was no difference in the average displacement between of HSPCs between non-irradiated versus irradiated mice (Figure 4B). At day 4 post-transplantation, HSPCs were more motile in non-conditioned NSG and NSG-hmSCF compared with irradiated mice (Figure 4C). However, looking more closely at the displacement values from non-irradiated recipients at day 4 (Figure 4C), it appears that there are two subpopulations with around half of the cells having a similar low displacement to irradiated mice and the remaining cells being motile. This is significant since there is around a 50% reduction in engraftment in non-irradiated recipients (Figure 4A). We suggest that fewer available niches in non-irradiated mice might explain these results. Thus, around half of the cells stop moving since they have found an empty niche and are able to contribute to engraftment, while the remaining cells are unable to find an empty niche and keep moving and do not receive the cues necessary to survive and therefore do not engraft. We nevertheless could not exclude that some microenvironmental changes occur between days 3 and 4 in conditioned mice, which are responsible for this change in motility of HPCs. Further analysis using new mouse models not requiring conditioning to achieve comparable engraftment levels will definitively address whether the increase in available niches correlates with an increase in less motile HSCs (similarly to conditioning), as in these mice more niche space is available for transplanted HSPCs due to functional impairment of endogenous HSPCs (Cosgun et al., 2014).

In order to determine whether there are any gene changes in transplanted +/− cells between 3 and 4 days after transplantation that would account for a decrease in motility, for example, by increased adhesion, we performed microarray analysis on +/− and +/+ cells that had been CFSE labeled and transplanted into sublethally irradiated NSG mice and recovered them from the BM by FACS sorting at 3 and 4 days after transplantation. However, we did not detect any upregulation of genes mediating adhesion such as integrins and cadherins at day 4 that would explain why +/− cells stop moving (data not shown). This is likely because the function of adhesion molecules is not regulated at the gene transcription level and/or that adhesion molecule function is stable after transplantation and HSCs keep moving until they find an empty niche that expresses the correct receptor(s).

Disruption of Integrin Binding within the Niche 4 Days after Transplantation Reverses the HSPC-Specific Reduction in Motility 4 Days after Transplantation

When studying the movies of HSPCs migrating (Movies S1 and S2), we saw that while moving HSPCs continually changed shape but when stopped they remained rounded. In order for a cell to migrate it must polarize, which leads to changes in cell shape. We wondered whether +/− cells became less motile at day 4 after transplantation due to changes in their ability to polarize. One of the key players in cell polarization is *Rac1*. It was shown that deletion of *Rac1* in HSPCs inhibited their ability to form cobblestones in vitro and the frequency of $Rac1^{-/-}$ HSPCs that were able to home to and be retained in vivo at the endosteal surface was reduced (Cancelas et al., 2005). In order to determine whether altering RAC1-mediated polarization could change the motility of +/− cells at day 4, we used a RAC1-GEF specific inhibitor (NSC23766) in vivo. To do this, +/− cells were imaged 4 days after transplantation to confirm the expected pattern of +/− immobility. Afterward, NSC23766 was injected and imaging was continued for up to 4-hr post-injection. Surprisingly, there was no increase in displacement (Figure 5A), speed (Figure 5B), or straightness (Figure 5C) of +/− cells, and their position relative to the bone surface did not change (Figure 5D). However, there was a highly significant decrease in sphericity indicating that RAC1 treatment does affect polarization of +/− cells (Figure 5E). Thus, it appears that inhibiting polarization is not sufficient to change migration, and there are other molecules that affect motility, for instance adhesion molecules retain, or anchor, HSPCs in their niche.

One of the most extensively characterized signaling pathways in HSC homing is the CXCR4/SDF1 axis. CXCL12 binding to CXCR4 enhances the affinity of integrins, in particular VLA-4, which mediates HSC cell adhesion in the niche (Hartmann et al., 2008; Peled et al., 1999a, 2000). Impairment of integrin or CXCR4/SDF1 expression or neutralization of their function by antibody treatment is known to be associated with reduced HSC homing (Kollet et al., 2001; Peled et al., 1999b; Qian et al., 2006; Wagers et al., 2002). Treatment with the CXCR4 receptor antagonist, AMD3100, is a potent mobilizing agent (Broxmeyer et al., 2005; Liles et al., 2003), and inhibition of VLA-4 (via Bio5192, a small molecule inhibitor) alone and in combination with CXCR4 antagonists was shown to mobilize long-term HSPCs to the periphery more effectively than with AMD3100 alone (Bonig et al., 2009; Ramirez et al., 2009; Leone et al., 2003). However, the precise role of integrin(s) in the processes of extravasation from blood vessels into the BM niche versus their function in migration and lodgment in the niche is difficult to separate. In order to examine the role of VLA-4 specifically in the migration and lodgment of HSCs at day 4, we transplanted CFSE labeled cells in vivo. At 4 days later, cells were imaged to confirm the expected lack of migration of +/− cells and continued movement of +/+ cells. We then injected VLA-4 inhibitor (Bio 5192) and continued to track migration up to 4 hr after injection. Addition of Bio 5192 resulted in a significant increase in displacement (Figure 5F; Movie S5) and straightness (Figure 5H; Movie S5), a trend of, although not significant, increased distance to the bone surface (Figure 5I) and a significant decrease in sphericity (Figure 5J), indicating a role for VLA-4 in the non-motile phenotype of stem cells 4 days after transplantation. However, there was a subpopulation of cells that did not respond to this inhibition and remained relatively non-motile; thus, it is likely that other integrins also regulate HSPC adhesion in the niche. Since CXCR4/SDF1 might regulate the affinity of various integrins in addition to VLA-4, we treated cells with the CXCR4 antagonist, AMD3100. Similar to beta1 inhibition, AMD3100 treatment significantly increased the displacement (Figure 5F; Movie S6), speed (Figure 5G; Movie S6) and trend, but not significant, increase in straightness (Figure 5H; Movie S6) of the majority of +/− cells, their distance from the bone surface (Figure 5I), and significantly decreased sphericity of the cells (Figure 5J), although again there was still a subpopulation of cells that did not respond to treatment. It is likely that adhesion and retention in the niche are controlled by a complex array of adhesion molecules, extracellular matrix components, and that retention to specific stromal cell subtypes involves different molecules that are yet to be defined.

DISCUSSION

The location and composition of the HSC niche are areas of intense debate in the stem cell field with numerous studies

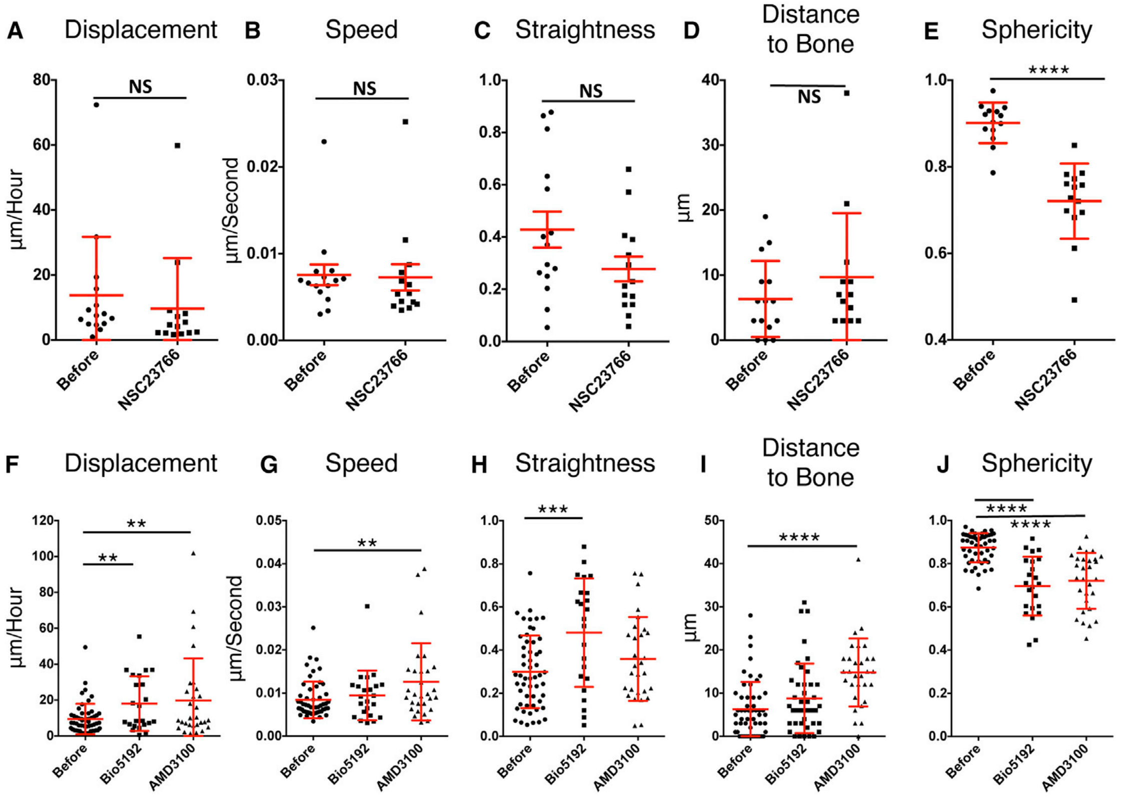

Figure 5. In Vivo Blocking of Integrins Reverses the Non-motile Phenotype of HSPCs

(A–E) In vivo treatment at day 4 post-transplantation of NSG mice with RAC1-GEF inhibitor NSC 23776 (3 mg/kg). Displacement (A), Speed (B), Straightness, (C) Distance to Bone, (D) and Sphericity (E) were measured from cells imaged in the calvaria, calculated before and after treatment. N = 2 recipients of six to nine pooled CB units for each recipient.

(F–J) In vivo treatment at day 4 post-transplantation of NSG mice with beta1 inhibitor-Bio 5192 (1 mg/kg) or CXCR4 antagonist-AMD3100 (1mg/kg). Displacement (F), speed (G), straightness (H), distance to bone (I), and sphericity (J) were measured from cells imaged in the calvaria and were calculated before and after treatment. N = 2 recipients (AMD3100) and 3 recipients (Bio 5192) of six to nine pooled CB units for each recipient. Error bars represent ± SD. $^{**}p < 0.01$, $^{***}p < 0.001$; $^{****}p < 0.0001$.

describing the niche in the mouse (Ellis et al., 2011; Kiel et al., 2005; Nombela-Arrieta et al., 2013; Zhang et al., 2003; Sugiyama et al., 2006; Méndez-Ferrer et al., 2010; Calvi et al., 2003), human trephine sections (Guezguez et al., 2013), and zebrafish (Tamplin et al., 2015). However, although a recent publication described changes in motility of HSPC in the context of parasitic infection (Rashidi et al., 2014), there is very little published information regarding the dynamics of HSPC migration in vivo and how this correlates with functionality. Dr. Nilsson's group reported that just 5 hr after mouse HSC transplantation, these cells can be further enriched functionally by extracting them from the endosteal region compared to the central bone region (Grassinger et al., 2010), demonstrating that phenotypically identical HSCs isolated from different regions of the BM had different biological potential. Our data add further information on the dynamic behavior of HSPCs by tracking human and mouse HSC and HPC populations in vivo and showing that there are significant differences in their motility. Specifically, we saw that HSCs exhibit a change in motility after transplantation to become less motile, whereas most HPCs remain motile. This could be due to a cell-intrinsic effect where cells are programmed to be less motile or due to cell extrinsic cues from the microenvironment that direct the cells to stop migrating. The probability of an HSC encountering the correct extrinsic signal increases over time, which may account for the length of time for the population as a whole to stop moving and why there are some non-motile cells at earlier time points. The motility data obtained 3 days after transplantation indicate that HSCs are capable of displacing equally as well as HPCs. Therefore, the latter suggestion is more likely, and once an HSC has found an empty niche that expresses the correct receptor/ligand pair, it adheres and is anchored at that position. Pharmacological blocking with inhibitors of VLA-4 and CXCR4 reversed the

non-motile phenotype of +/− cells, indicating that the anchor may be VLA-4, and other, integrins dependent. Interestingly, our results demonstrate that the dynamic behaviors that distinguish HSCs from HPCs are consistent in both mouse and human, indicating that motility behavior may be a way to discriminate between stem and progenitor cells. The differences in motility between stem and progenitor populations could be explained by differences in niche requirements between stem and progenitor cells; stem cells are dependent on the niche for survival, quiescence, and proliferation, while progenitors do not require the same niche interactions, if any, for their function.

Conventionally, the location of transplanted cells 16 hr after transplantation is used to describe the homing niche for HSPCs (Lapidot et al., 2005). However, the data presented here suggest that the position of HSCs at 16 hr after transplantation cannot be used to define the niche since cells are still migrating. Therefore, we suggest that lodgment of human HSCs be defined as their position when they stop moving. The only method to determine when a cell has stopped migrating is by intravital imaging of live, intact BM. At present, this is only possible in the calvaria. As previously reported, mouse HSCs in the skull and femurs are present at similar frequencies and are functionally identical (Lassailly et al., 2013), indicating the presence of a functional HSC niche in the calvaria. Since HSCs have not yet been imaged live in the long bones without first damaging the niche architecture (Köhler et al., 2009; Lewandowski et al., 2010; Xie et al., 2009) or at high resolution (Branchini et al., 2010) the calvaria represents the only BM cavity, which can be imaged non-invasively and with sufficient resolution to track single-cell migration and proliferation at this time. As indicated in our time-lapse imaging, HSCs continue to migrate after transplantation, and as such, it is difficult to pinpoint the location of the stem cell niche. The data show that one needs to answer questions regarding HSC homing and lodgment using multiple approaches, including live tracking analyses to determine when stem cells stop moving and the position of the cells to define where in the niche they lodge. Observing the effect on location and motility after targeting specific stromal cell populations and/or niche-hematopoietic signaling pathways should help further characterize the human HSC niche.

EXPERIMENTAL PROCEDURES

Labeling, Transplantation, and Imaging of HSC/HPCs for Tracking

Human HSPCs were obtained from cord blood (CB) and mouse HSPCs obtained from C57BL/6 BM. For further details on human and mouse HSPC purification, labeling, and transplantation, please see Supplemental Experimental Procedures.

RAC1, Beta1 Inhibition, and CXCR4 Antagonism of HSC Motility

To determine the effect of inhibitors on cell migration, +/− cells were transplanted into sublethally irradiated NSG recipients. At 4 days after transplantation, cells were imaged by intravital microscopy. After 2 to 3 hr of imaging, NSC 23766 (3 mg/kg, Sigma), Bio 5192 (1 mg/kg, Tocris), or AMD3100 (1 mg/kg, Sigma) were injected IV into the mouse, and imaging was resumed 30 min later for up to 4-hr post-injection.

Image Analysis and Cell Tracking

Each z stack of images is rendered in 3D using Imaris software (Bitplane). To correct for drift or breathing artifacts, a spot is defined that does not move, and all time points are corrected to this spot. An iso-surface is generated for bone, and spot function is used to find a cell(s) of interest. The distance between center of the cell and closest pixel of bone surface is calculated using Imaris XT extension (Bitplane), and results are entered into Prism (GraphPad Software). The mean is highlighted with a red horizontal line, and error bars represent ± SD. The Shapiro-Wilk test was performed to assess normality of raw and log-transformed data. Two-way ANOVA with interaction term was performed to assess differences between groupings where two variables (phenotype and time point) exist. In experiments that compared one variable, t test was used to compare differences. Statistical analyses were performed using R 3.0.2.

To calculate displacement, the distance between the start and end point of each migration track in a straight line was calculated. Each track was corrected by time to give displacement per hour. Displacement in a straight line from start and end points was used in preference to track length because a cell moving randomly with no overall movement can have the same track length as a cell moving over a larger distance in a straight line. In-built algorithms generate speed and straightness measurements. Straightness describes whether the cell moves in a straight line (a value close to 1) or in a random direction (a value close to 0). Actual migration tracks were generated by the “Tube Plot” algorithm in MatLab (Wolfram Mathmatica).

Sphericity was calculated by measuring the area and perimeter of a single section through the center of a cell using this equation:

$$x = (4 \times \text{Area} \times \pi)/(\text{Perimeter}\hat{\ }2).$$

Further information can be found in Supplemental Experimental Procedures.

SUPPLEMENTAL INFORMATION

Supplemental Information includes Supplemental Experimental Procedures, Figures S1 and S2, and Movies S1–S6 and can be found with this article online at http://dx.doi.org/10.1016/j.stemcr.2015.09.003.

ACKNOWLEDGMENTS

We thank staff in the Biological Resource Unit, Flow Cytometry, In Vivo Imaging, and Light Microscopy core facilities at the London Research Institute for their valuable help. We specifically thank Dr. Stuart Horswell from the bioinformatics and for his help with

statistical analysis. We also thank Mrs. Shohreh Beski (Obstetric Department, Royal London Hospital, London, UK) and her team as well as Mrs. Jashu Patel for helping in the collection of CB used in this study. This work was funded by Cancer Research UK.

Received: May 12, 2015
Revised: September 8, 2015
Accepted: September 8, 2015
Published: October 8, 2015

REFERENCES

Bonig, H., Watts, K.L., Chang, K.H., Kiem, H.P., and Papayannopoulou, T. (2009). Concurrent blockade of alpha4-integrin and CXCR4 in hematopoietic stem/progenitor cell mobilization. Stem Cells *27*, 836–837.

Branchini, B.R., Ablamsky, D.M., Davis, A.L., Southworth, T.L., Butler, B., Fan, F., Jathoul, A.P., and Pule, M.A. (2010). Red-emitting luciferases for bioluminescence reporter and imaging applications. Anal. Biochem. *396*, 290–297.

Brehm, M.A., Racki, W.J., Leif, J., Burzenski, L., Hosur, V., Wetmore, A., Gott, B., Herlihy, M., Ignotz, R., Dunn, R., et al. (2012). Engraftment of human HSCs in nonirradiated newborn NOD-scid IL2rγ null mice is enhanced by transgenic expression of membrane-bound human SCF. Blood *119*, 2778–2788.

Broxmeyer, H.E., Orschell, C.M., Clapp, D.W., Hangoc, G., Cooper, S., Plett, P.A., Liles, W.C., Li, X., Graham-Evans, B., Campbell, T.B., et al. (2005). Rapid mobilization of murine and human hematopoietic stem and progenitor cells with AMD3100, a CXCR4 antagonist. J. Exp. Med. *201*, 1307–1318.

Calvi, L.M., Adams, G.B., Weibrecht, K.W., Weber, J.M., Olson, D.P., Knight, M.C., Martin, R.P., Schipani, E., Divieti, P., Bringhurst, F.R., et al. (2003). Osteoblastic cells regulate the haematopoietic stem cell niche. Nature *425*, 841–846.

Cancelas, J.A., Lee, A.W., Prabhakar, R., Stringer, K.F., Zheng, Y., and Williams, D.A. (2005). Rac GTPases differentially integrate signals regulating hematopoietic stem cell localization. Nat. Med. *11*, 886–891.

Cosgun, K.N., Rahmig, S., Mende, N., Reinke, S., Hauber, I., Schäfer, C., Petzold, A., Weisbach, H., Heidkamp, G., Purbojo, A., et al. (2014). Kit regulates HSC engraftment across the human-mouse species barrier. Cell Stem Cell *15*, 227–238.

De Clerck, L.S., Bridts, C.H., Mertens, A.M., Moens, M.M., and Stevens, W.J. (1994). Use of fluorescent dyes in the determination of adherence of human leucocytes to endothelial cells and the effect of fluorochromes on cellular function. J. Immunol. Methods *172*, 115–124.

Ellis, S.L., Grassinger, J., Jones, A., Borg, J., Camenisch, T., Haylock, D., Bertoncello, I., and Nilsson, S.K. (2011). The relationship between bone, hemopoietic stem cells, and vasculature. Blood *118*, 1516–1524.

Grassinger, J., Haylock, D.N., Williams, B., Olsen, G.H., and Nilsson, S.K. (2010). Phenotypically identical hemopoietic stem cells isolated from different regions of bone marrow have different biologic potential. Blood *116*, 3185–3196.

Guezguez, B., Campbell, C.J., Boyd, A.L., Karanu, F., Casado, F.L., Di Cresce, C., Collins, T.J., Shapovalova, Z., Xenocostas, A., and Bhatia, M. (2013). Regional localization within the bone marrow influences the functional capacity of human HSCs. Cell Stem Cell *13*, 175–189.

Hartmann, T.N., Grabovsky, V., Pasvolsky, R., Shulman, Z., Buss, E.C., Spiegel, A., Nagler, A., Lapidot, T., Thelen, M., and Alon, R. (2008). A crosstalk between intracellular CXCR7 and CXCR4 involved in rapid CXCL12-triggered integrin activation but not in chemokine-triggered motility of human T lymphocytes and CD34+ cells. J. Leukoc. Biol. *84*, 1130–1140.

Kiel, M.J., Yilmaz, O.H., Iwashita, T., Yilmaz, O.H., Terhorst, C., and Morrison, S.J. (2005). SLAM family receptors distinguish hematopoietic stem and progenitor cells and reveal endothelial niches for stem cells. Cell *121*, 1109–1121.

Kim, M., Cooper, D.D., Hayes, S.F., and Spangrude, G.J. (1998). Rhodamine-123 staining in hematopoietic stem cells of young mice indicates mitochondrial activation rather than dye efflux. Blood *91*, 4106–4117.

Köhler, A., Schmithorst, V., Filippi, M.D., Ryan, M.A., Daria, D., Gunzer, M., and Geiger, H. (2009). Altered cellular dynamics and endosteal location of aged early hematopoietic progenitor cells revealed by time-lapse intravital imaging in long bones. Blood *114*, 290–298.

Kollet, O., Spiegel, A., Peled, A., Petit, I., Byk, T., Hershkoviz, R., Guetta, E., Barkai, G., Nagler, A., and Lapidot, T. (2001). Rapid and efficient homing of human CD34(+)CD38(−/low)CXCR4(+) stem and progenitor cells to the bone marrow and spleen of NOD/SCID and NOD/SCID/B2m(null) mice. Blood *97*, 3283–3291.

Lapidot, T., Dar, A., and Kollet, O. (2005). How do stem cells find their way home? Blood *106*, 1901–1910.

Lassailly, F., Griessinger, E., and Bonnet, D. (2010). "Microenvironmental contaminations" induced by fluorescent lipophilic dyes used for noninvasive in vitro and in vivo cell tracking. Blood *115*, 5347–5354.

Lassailly, F., Foster, K., Lopez-Onieva, L., Currie, E., and Bonnet, D. (2013). Multimodal imaging reveals structural and functional heterogeneity in different bone marrow compartments: functional implications on hematopoietic stem cells. Blood *122*, 1730–1740.

Leone, D.R., Giza, K., Gill, A., Dolinski, B.M., Yang, W., Perper, S., Scott, D.M., Lee, W.C., Cornebise, M., Wortham, K., et al. (2003). An assessment of the mechanistic differences between two integrin alpha 4 beta 1 inhibitors, the monoclonal antibody TA-2 and the small molecule BIO5192, in rat experimental autoimmune encephalomyelitis. J. Pharmacol. Exp. Ther. *305*, 1150–1162.

Lewandowski, D., Barroca, V., Ducongé, F., Bayer, J., Van Nhieu, J.T., Pestourie, C., Fouchet, P., Tavitian, B., and Roméo, P.H. (2010). In vivo cellular imaging pinpoints the role of reactive oxygen species in the early steps of adult hematopoietic reconstitution. Blood *115*, 443–452.

Liles, W.C., Broxmeyer, H.E., Rodger, E., Wood, B., Hübel, K., Cooper, S., Hangoc, G., Bridger, G.J., Henson, G.W., Calandra, G., and Dale, D.C. (2003). Mobilization of hematopoietic

progenitor cells in healthy volunteers by AMD3100, a CXCR4 antagonist. Blood *102*, 2728–2730.

Lo Celso, C., Fleming, H.E., Wu, J.W., Zhao, C.X., Miake-Lye, S., Fujisaki, J., Côté, D., Rowe, D.W., Lin, C.P., and Scadden, D.T. (2009). Live-animal tracking of individual haematopoietic stem/progenitor cells in their niche. Nature *457*, 92–96.

Méndez-Ferrer, S., Michurina, T.V., Ferraro, F., Mazloom, A.R., Macarthur, B.D., Lira, S.A., Scadden, D.T., Ma'ayan, A., Enikolopov, G.N., and Frenette, P.S. (2010). Mesenchymal and haematopoietic stem cells form a unique bone marrow niche. Nature *466*, 829–834.

Morrison, S.J., and Scadden, D.T. (2014). The bone marrow niche for haematopoietic stem cells. Nature *505*, 327–334.

Nolte, M.A., Kraal, G., and Mebius, R.E. (2004). Effects of fluorescent and nonfluorescent tracing methods on lymphocyte migration in vivo. Cytometry Part A *61*, 35–44.

Nombela-Arrieta, C., Pivarnik, G., Winkel, B., Canty, K.J., Harley, B., Mahoney, J.E., Park, S.Y., Lu, J., Protopopov, A., and Silberstein, L.E. (2013). Quantitative imaging of haematopoietic stem and progenitor cell localization and hypoxic status in the bone marrow microenvironment. Nat. Cell Biol. *15*, 533–543.

Notta, F., Doulatov, S., Laurenti, E., Poeppl, A., Jurisica, I., and Dick, J.E. (2011). Isolation of single human hematopoietic stem cells capable of long-term multilineage engraftment. Science *333*, 218–221.

Oguro, H., Ding, L., and Morrison, S.J. (2013). SLAM family markers resolve functionally distinct subpopulations of hematopoietic stem cells and multipotent progenitors. Cell Stem Cell *13*, 102–116.

Peled, A., Grabovsky, V., Habler, L., Sandbank, J., Arenzana-Seisdedos, F., Petit, I., Ben-Hur, H., Lapidot, T., and Alon, R. (1999a). The chemokine SDF-1 stimulates integrin-mediated arrest of CD34(+) cells on vascular endothelium under shear flow. J. Clin. Invest. *104*, 1199–1211.

Peled, A., Petit, I., Kollet, O., Magid, M., Ponomaryov, T., Byk, T., Nagler, A., Ben-Hur, H., Many, A., Shultz, L., et al. (1999b). Dependence of human stem cell engraftment and repopulation of NOD/SCID mice on CXCR4. Science *283*, 845–848.

Peled, A., Kollet, O., Ponomaryov, T., Petit, I., Franitza, S., Grabovsky, V., Slav, M.M., Nagler, A., Lider, O., Alon, R., et al. (2000). The chemokine SDF-1 activates the integrins LFA-1, VLA-4, and VLA-5 on immature human CD34(+) cells: role in transendothelial/stromal migration and engraftment of NOD/SCID mice. Blood *95*, 3289–3296.

Qian, H., Tryggvason, K., Jacobsen, S.E., and Ekblom, M. (2006). Contribution of alpha6 integrins to hematopoietic stem and progenitor cell homing to bone marrow and collaboration with alpha4 integrins. Blood *107*, 3503–3510.

Ramirez, P., Rettig, M.P., Uy, G.L., Deych, E., Holt, M.S., Ritchey, J.K., and DiPersio, J.F. (2009). BIO5192, a small molecule inhibitor of VLA-4, mobilizes hematopoietic stem and progenitor cells. Blood *114*, 1340–1343.

Rashidi, N.M., Scott, M.K., Scherf, N., Krinner, A., Kalchschmidt, J.S., Gounaris, K., Selkirk, M.E., Roeder, I., and Lo Celso, C. (2014). In vivo time-lapse imaging shows diverse niche engagement by quiescent and naturally activated hematopoietic stem cells. Blood *124*, 79–83.

Sugiyama, T., Kohara, H., Noda, M., and Nagasawa, T. (2006). Maintenance of the hematopoietic stem cell pool by CXCL12-CXCR4 chemokine signaling in bone marrow stromal cell niches. Immunity *25*, 977–988.

Takagi, S., Saito, Y., Hijikata, A., Tanaka, S., Watanabe, T., Hasegawa, T., Mochizuki, S., Kunisawa, J., Kiyono, H., Koseki, H., et al. (2012). Membrane-bound human SCF/KL promotes in vivo human hematopoietic engraftment and myeloid differentiation. Blood *119*, 2768–2777.

Tamplin, O.J., Durand, E.M., Carr, L.A., Childs, S.J., Hagedorn, E.J., Li, P., Yzaguirre, A.D., Speck, N.A., and Zon, L.I. (2015). Hematopoietic stem cell arrival triggers dynamic remodeling of the perivascular niche. Cell *160*, 241–252.

Wagers, A.J., Allsopp, R.C., and Weissman, I.L. (2002). Changes in integrin expression are associated with altered homing properties of Lin(−/lo)Thy1.1(lo)Sca-1(+)c-kit(+) hematopoietic stem cells following mobilization by cyclophosphamide/granulocyte colony-stimulating factor. Exp. Hematol. *30*, 176–185.

Xie, Y., Yin, T., Wiegraebe, W., He, X.C., Miller, D., Stark, D., Perko, K., Alexander, R., Schwartz, J., Grindley, J.C., et al. (2009). Detection of functional haematopoietic stem cell niche using real-time imaging. Nature *457*, 97–101.

Zhang, J., Niu, C., Ye, L., Huang, H., He, X., Tong, W.G., Ross, J., Haug, J., Johnson, T., Feng, J.Q., et al. (2003). Identification of the haematopoietic stem cell niche and control of the niche size. Nature *425*, 836–841.

Stem Cell Reports, Volume *5*

Supplemental Information

Different Motile Behaviors of Human Hematopoietic Stem versus Progenitor Cells at the Osteoblastic Niche

Katie Foster, François Lassailly, Fernando Anjos-Afonso, Erin Currie, Kevin Rouault-Pierre, and Dominique Bonnet

Supplemental Figure 1

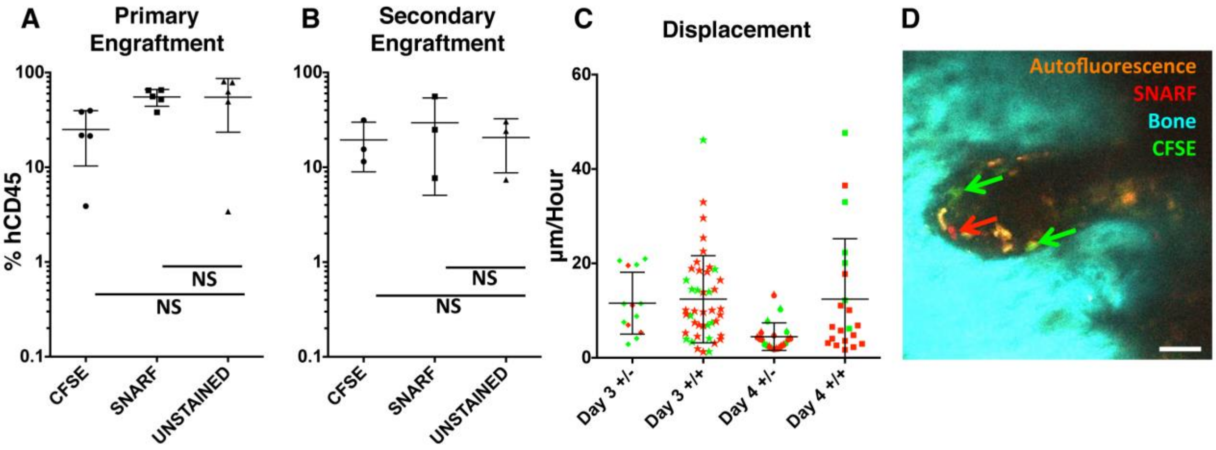

Supplemental Figure 1 Legend:

Cell labeling and Image analysis protocol, related to Figure 1. (A & B) Umbilical cord blood mononuclear cells were lineage depleted, and 100,000 Lin^{-} cells were injected IV into the tail vein of 3.75Gy sub-lethally irradiated NSG recipients, N = 5 CFSE recipients, N = 5 SNARF recipients and N = 5 unstained recipients. After 12 weeks, BM was extracted and an aliquot analyzed by flow cytometry to measure the engraftment of human cells (% Human $CD45^{+}$). The remaining cells from the 5 recipients per group were pooled and depleted for mouse CD45 cells and transplanted by IV injection into the tail vein of three secondary 3.75Gy sub-lethally irradiated NSG mice, N = 3 CFSE recipients, N = 3 SNARF recipients and N = 3 unstained recipients. Error bars represent +/- SD *$p < 0.05$, **$p < 0.01$, ***$p < 0.001$. (C) Displacement per hour of +/+ and +/- cells at day 3 and 4 taken from Figure 3Ai. Cells that were stained with CFSE are colored in green and cells that were labeled with SNARF are shown in red. (D) Representative image of CFSE and SNARF labeled cells in the calvaria to show specificity of each dye (only present in the green or red channel, see arrows) and auto-fluorescence, which appears in both channels and thus is colored yellow/orange. Objects, which appear in both channels, are excluded from further analysis. Scale bar = 50um.

Supplemental Figure 2

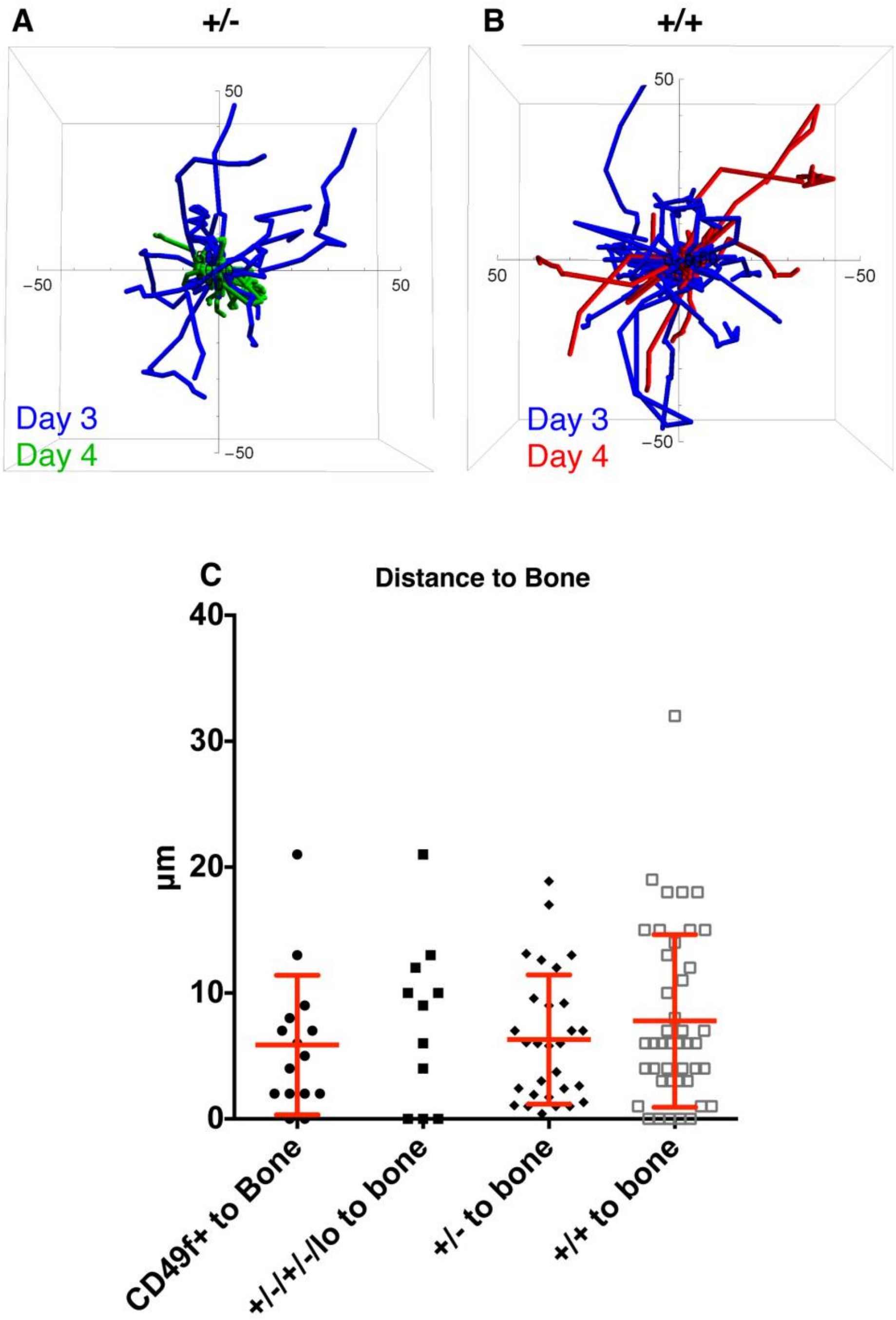

Supplemental Figure 2 Legend:

Extended *in vivo* tracking plots of human HSPCs (related to Figure 3). Umbilical cord blood mononuclear cells were lineage depleted, stained with antibodies recognizing Lin, CD38 and CD34 and flow sorted to purify $Lin^-CD34^+CD38^-$ (+/-) and $Lin^-CD34^+CD38^+$ (+/+) populations. The cells were labeled with cell dyes and injected into 3.75Gy sub lethally irradiated NSG recipients. (A) The track plot of +/- cells at day 3 (blue) and day 4 (green) are overlaid to show the difference in motility between the two time points. This is the same data as shown in Figure 3D with Day 3 and 4 tracks shown on the same plot rather than +/- and +/+ tracks on the same plot. Total track length of cells in Figure 3A, which varies from 1-2 hours in length. (B) Track plots of +/+ cells at day 3 (blue) and day 4 (red) are overlaid to show a similar pattern of motility at the two time points. This is the same data as shown in Figure 3D with Day 3 and 4 tracks shown on the same plot rather than +/- and +/+ tracks on the same plot. Total track length of cells in Figure 3A, which varies from 1-2 hours in length. (C) Umbilical cord blood mononuclear cells were lineage depleted, stained with antibodies recognizing CD38, CD34, CD45RA, CD90 and CD49f or the cell dye Rhodamine123 and flow sorted to purify HSCs stained with CFSE and transplanted into sub-lethally irradiated NSG mice. Four days after transplantation, the distance between CFSE labeled $CD49f^+$, +/-/+/-/lo, +/+ and +/- cells and both bone surface was calculated. N = 3 recipients of $CD49f^+$ cells, N = 2 recipients of +/-/+/-/lo cells and N = 3 recipients of +/- and +/+ cells from approximately 6-9 pooled CB units for each recipient. Error bars represent +/- SD.

Supplemental Experimental Procedures

Mice

All animal procedures were performed in compliance with Cancer Research UK (CRUK) animal ethics committee and UK Home Office guidelines. NOD-SCID IL2Rg^{null} (NSG) and C57BL/6 strains (All Jackson Laboratory, Bar Harbor, Maine, USA), were bred at CRUK's Biological Resources Unit in individually vented cages (IVCs) under Specific Pathogen Free (SPF) conditions. Sub-lethal irradiation was carried out at 3.75Gy with a 137Cs 637 irradiator.

Human HSPCs

Umbilical cord blood was obtained from the Royal London Hospital (London, UK) after informed consent via a protocol approved by the East London Research Ethics Committee and carried out in accordance with the principles of the Helsinki declaration. Mononuclear cells (MNCs) were obtained by centrifugation over a Ficoll-paque gradient (GE Healthcare) and mature cells removed by immunomagnetic depletion using StemSep® Human Progenitor enrichment cocktail (Stem Cell Technologies, Vancouver, Canada). Lineage negative cells were stained with antibodies against Lin, CD34 and CD38 (Clone HB-7) to define HSC- and HPC-enriched populations and further with CD45RA, CD90, CD49f (all BD Biosciences) and the dye Rhodamine123 (Thermo Fisher Scientific~~Life Technologies~~) to obtain highly purified HSCs and sorted by flow cytometric activated cell sorting (FACS).

Mouse HSPCs

C57Bl/6 BM was flushed from femurs and tibias and through crushing of iliac crests and vertebrae in phosphate-buffered saline (PBS) with 2% fetal calf serum (FCS, PAA Labs), heparin and DNAse (Sigma Aldrich). Red blood cells were lysed and nucleated cells were depleted of mature cells using EasySep Mouse Hematopoietic Progenitor kit (Stem Cell

Technologies). Lineage depleted cells were stained with Lin, Sca1, cKit, CD150 and CD48 (BD Biosciences) before FACS sorting.

Labeling, transplantation and imaging of HSC/HPCs for tracking

Following purification, HSPCs were washed twice with PBS before incubating with 2 microM of CFSE for 10 minutes, •••••• microM of•••••• for 20 minutes at 37°C (both dyes from Thermo Fisher Scientific~~Life Technologies, UK~~). After incubation, 1ml of ice-cold serum was added before washing and suspending in 2% FCS in PBS. Approximately 2x10^{5} Lin^{-}/CD34^{+}/CD38^{-} (+/-) and 1x10^{6} Lin^{-}/CD34^{+}/CD38^{+} (+/+), 1x10^{5} Lin^{-}/CD34^{+}/CD38^{-}/CD90^{+}/CD45RA^{-}/Rhodamine-Low (+/-/+/-/lo) cells, 5x10^{4} Lin^{-}CD34^{+}/CD38^{-}/CD90^{+}/CD45RA^{-}/CD49f^{+} (CD49f^{+}), 2.5x10^{4} Lin^{-}/Sca1^{+}/cKit^{+}/CD48^{-}/CD150^{+} (LSK SLAM^{+}), 2.5x10^{5} Lin^{-}/Sca1^{+}/cKit^{+}/ not CD48^{-}CD150^{+} (LSK SLAM^{-}), cells per recipient were transplanted IV into 3.75Gy irradiated NSG. Identical numbers of stem-enriched and progenitors were not injected because the homing of progenitors is less efficient than stem-enriched populations. Immediately prior to imaging, streptavidin conjugated Quantum dots (Qdots)-655 (Thermo Fisher Scientific~~Life Technologies~~) were IV injected to label endothelial cells. Mice were anesthetized with 2.5% Isoflurane, the head shaved and held in a stereotaxic skull holder. A skin incision revealed the calvaria and methylcellulose (4%, Sigma Aldrich) was applied to prevent the tissue drying. Each mouse was imaged for 4-6 hours before culling, and a separate recipient was used for each time-point. Images were obtained on a Zeiss 710 NLO laser scanning multiphoton microscope with a 20x 1.0 NA water immersion lens. The microscope is equipped with a MaiTai “High Performance” fully automated 1-box mode-locked Ti:Sapphire laser with DeepSee dispersion compensation (Spectra-Physics), tuned to 800nm excitation wavelength. Bone signal- (Second Harmonic Generation signal) was collected at 380-485nm, CFSE at 500-550nm, SNARF at 555-625 and qDots-655 at 640-690nm by non-descanned detectors.

In vivo assays to assess the impact of cell dyes on hematopoiesis

To assess engraftment, 100,000 Lin$^-$ cells stained with CFSE or SNARF and unstained cells were injected intra-bone into 3.75Gy irradiated NSG mice. After 12 weeks, BM was extracted and stained with antibodies recognizing human CD45-PeCy7, mouse CD45-PerCP, human CD3-FITC, human CD19-APC and human CD33-PE (All BD Biosciences) and sorted by flow cytometry. Sorted human cells were IV injected into secondary NSG mice. After a further 12 weeks, BM was analyzed by flow cytometry using the same antibodies previously used to assess primary engraftment.

In vitro colony forming assay and _In vivo_ Limiting Dilution Assay (LDA) to assess functionality of transplanted stem cells

To estimate colony-forming ability, CFSE$^+$ cells were FACS sorted and embedded into methylcellulose with SCF, GM-CSF, IL-3 and EPO (H4434, Stem Cell Technologies). In addition, remaining CFSE labeled +/- sorted cells were injected IV into one irradiated NSG mice. After transplantation, BM was extracted from femurs, tibias and iliac crests and CFSE$^+$ cells re-sorted before adding to methylcellulose. Colonies were counted, washed and re-plated to generate secondary colonies. To estimate stem cell frequency, freshly sorted CFSE labeled +/- cells ("before transplantation") and cells extracted from the BM of mice transplanted with CFSE labeled +/- cells ("after transplantation") were injected intra-bone into sublethally irradiated NSG recipients. Engraftment was assessed by FACS 12 weeks after transplantation and was scored positive if BM contained multilineage engraftment above 0.1%. LDA was calculated with ELDA: Extreme Limiting Dilution Assay software (Hu and Smyth, 2009).

Table of all antibodies used in this study

Antibody Type	Clone	Company	Cat number

Human Lin FITC	Mix of different Ab	BDbiosciences	340456
Human CD34 APC	581	BDbiosciences	555824
Human CD38 PE	HB7	BDbiosciences	345806
Human CD38 PeCy7	HB7	BDbiosciences	335825
Human CD45 PeCy7	HI30	eBiosciences	557748
Human CD3 FITC	HIT3a	BDbiosciences	555339
Human CD33 PE	WM53	BDbiosciences	555450
Human CD19 APC	HIB19	BDbiosciences	555415
Human CD49f	GoH3	BDbiosciences	555736
Human CD45RA FITC	HI100	BDbiosciences	555489
Human CD90 APC	5E10	eBiosciences	17-0900-42
Mouse CD45 PerCP	30-F11	BDbiosciences	557235
Mouse CD45 PeCy5	30-F11	BDbiosciences	553082
Mouse CD150	TC15-12F12.2	Biolegend	115912
Mouse CD48 FITC	HM48-1	BDbiosciences	557484
Mouse cKIT APC	2B8	eBiosciences	1171-83
Mouse SCA1 PeCy7	D7	eBiosciences	25-5982-82
Mouse Lin	Mix of different Ab	StemCell Technologies	19756
StreptAvidin PerCP	-	BDbiosciences	554064

Additional References

Hu, Y., and Smyth, G.K. (2009). ELDA: extreme limiting dilution analysis for comparing depleted and enriched populations in stem cell and other assays. Journal of immunological methods *347*, 70-78.

Video Legends

Video 1. A single z-section showing Human +/- (green) and +/+ (red) cell migration at day 3 after transplantation. Endothelial cells on blood vessels are shown in magenta. Scale bar = 40μm.

Video 2. A single z-section showing Human +/- (green) and +/+ (red) cell migration at day 4 after transplantation. Endothelial cells on blood vessels are shown in magenta, and the bone surface in cyan. Scale bar = 50μm.

Video 3. A single z-section showing a Mouse SLAM^{+} cell (green) migrating at 16 hours after transplantation. Endothelial cells on blood vessels are shown in magenta and the bone surface in cyan. Scale bar = 50μm.

Video 4. A single z-section showing a Mouse LSK^{+} cell (red) migrating at 16 hours after transplantation. Endothelial cells on blood vessels are shown in magenta and the bone surface in cyan. Scale bar = 40μm.

Video 5. A single z-section showing a human +/- cells in green 4 days after transplantation and IV injection of Bio5192. The bone surface is shown in cyan and auto-fluorescence in orange. Scale bar = 40μm.

Video 6. A single z-section showing human +/- cells 4 days after transplantation and IV injection of AMD3100. Endothelial cells are shown in magenta. Scale bar = 40μm.
